# Supplementary material for: Characterising the patient experience of diagnostic lumbar puncture in idiopathic intracranial hypertension: a cross-sectional online survey
Source: BMJ Open. 2018 May 30;8(5):e020445. doi: 10.1136/bmjopen-2017-020445 (PMC5988086; doi:10.1136/bmjopen-2017-020445)
Supplement: Supplementary file 1 [file bmjopen-2017-020445supp001.pdf]

IIHUK Online Questionnaire

Demographics

1. How old were you at time of diagnostic LP?
2. What was date of your first lumbar puncture? (Do not worry if you cannot remember the exact day, but please specify month and year)
3. Since your first diagnostic LP, how many LPs have you had? (Please approximate if not sure of the exact number)
4. What is your height (metres)?
5. What is your weight (kg)?

Details of lumbar puncture

1. Was your first admission regarding your LP planned (you were sent an appointment by your doctor) or as an emergency?
2. Where in the hospital did the first LP take place? (Emergency department, Ward or In Theatre)
3. How many different doctors attempted your diagnosing LP? (i.e how many different doctors tried inserting a needle)
4. How many attempts were made to get a diagnostic LP? (An attempt being defined as a needle being inserted)
5. If the initial LP failed to get a reading did you go on to have it done in theatre under X-ray guidance?

Patient experience of lumbar puncture

1. How frightened were you about the thought of the LP before the procedure on a scale 0-10? (0 being not frightened at all, 10 being the most scared you’ve ever felt)
2. How well do you feel you were informed about the procedure prior to the LP on a scale 0-10? (0 being not informed, 10 being fully informed)
3. Was adequate analgesia (i.e. local anaesthetic) used in your diagnosing LP?

4. How much pain did you experience during the LP on a scale of 0-10? (0 being pain free, 10 being worst pain ever experienced)
5. On a scale of 0-10, how anxious do you feel about having LPs in the future? (0 being not anxious, 10 being the most anxious you've ever felt)
