# Supplementary material for: Characterising the patient experience of diagnostic lumbar puncture in idiopathic intracranial hypertension: a cross-sectional online survey
Source: BMJ Open. 2018 May 30;8(5):e020445. doi: 10.1136/bmjopen-2017-020445 (PMC5988086; doi:10.1136/bmjopen-2017-020445)
Supplement: Author's manuscript [file bmjopen-2017-020445.draft_revisions.pdf]

BMJ Open is committed to open peer review. As part of this commitment we make the peer review history of every article we publish publicly available.

When an article is published we post the peer reviewers' comments and the authors' responses online. We also post the versions of the paper that were used during peer review. These are the versions that the peer review comments apply to.

The versions of the paper that follow are the versions that were submitted during the peer review process. They are not the versions of record or the final published versions. They should not be cited or distributed as the published version of this manuscript.

BMJ Open is an open access journal and the full, final, typeset and author-corrected version of record of the manuscript is available on our site with no access controls, subscription charges or pay-per-view fees (http://bmjopen.bmj.com).

If you have any questions on BMJ Open's open peer review process please email info.bmjopen@bmj.com

Characterising the patient experience of diagnostic lumbar puncture in idiopathic intracranial hypertension; a cross-sectional online survey.

Journal:	*BMJ Open*
Manuscript ID	bmjopen-2017-020445
Article Type:	Research
Date Submitted by the Author:	06-Nov-2017
Complete List of Authors:	Scotton, William; University of Birmingham, Institute of Metabolism and Systems Research; University Hospitals Birmingham NHS Foundation Trust, Department of Neurology<br>Mollan, Susan; Institute of Metabolism and Systems Research, College of Medical and Dental Sciences, University of Birmingham; Birmingham Neuro-Ophthalmology Unit, Ophthalmology Department, University Hospitals Birmingham NHS Trust, Queen Elizabeth Hospital Birmingham<br>Walters, Thomas; Institute of Metabolism and Systems Research, College of Medical and Dental Sciences, University of Birmingham<br>Doughty, Sandra; Idiopathic Intracranial Hypertension UK<br>Botfield, Hannah; Institute of Metabolism and Systems Research, College of Medical and Dental Sciences, University of Birmingham; Centre for Endocrinology, Diabetes and Metabolism, Birmingham Health Partners<br>Markey, Keira; Institute of Metabolism and Systems Research, College of Medical and Dental Sciences, University of Birmingham; University Hospitals Birmingham NHS Foundation Trust, Department of Neurology<br>Yiangou, Andreas; Institute of Metabolism and Systems Research, College of Medical and Dental Sciences, University of Birmingham; University Hospitals Birmingham NHS Foundation Trust, Department of Neurology<br>Williamson, Shelley; Idiopathic Intracranial Hypertension UK<br>Sinclair, AJ; Institute of Metabolism and Systems Research, College of Medical and Dental Sciences, University of Birmingham; Centre for Endocrinology, Diabetes and Metabolism, Birmingham Health Partners
Keywords:	Adult neurology < NEUROLOGY, Neuro-ophthalmology < NEUROLOGY, NEUROPATHOLOGY, Neuro-ophthalmology < OPHTHALMOLOGY

Title: Characterising the patient experience of diagnostic lumbar puncture in idiopathic intracranial hypertension; a cross-sectional online survey.

Authors: William J. Scotton[1,2], Susan P. Mollan[1,3], Thomas Walters[1], Sandra Doughty[4], Hannah Botfield[1,5], Keira Markey[1,2], Andreas Yiangou[1,2], *Shelly Williamson[4], *Alexandra J. Sinclair[1,5].

Institutions

1. Institute of Metabolism and Systems Research, College of Medical and Dental Sciences, University of Birmingham, Birmingham, B15 2TT, United Kingdom
2. Department of Neurology, University Hospitals Birmingham NHS Foundation Trust, B15 2TH, United Kingdom.
3. Birmingham Neuro-Ophthalmology Unit, Ophthalmology Department, University Hospitals Birmingham NHS Trust, Queen Elizabeth Hospital Birmingham, Birmingham, UK
4. Idiopathic Intracranial Hypertension UK, Registered Charity in England & Wales No. 1143522 & Scotland SC043294
5. Centre for Endocrinology, Diabetes and Metabolism, Birmingham Health Partners, B15 2TH, United Kingdom

*share joint senior author

For peer review only

Word Count Abstract: 300

Word Count Paper: 2141 (not including 67 recommendations, 111 strengths/weaknesses)

Table(s): 1

Figure(s): 3

Corresponding author:

Alexandra Sinclair, Institute of Metabolism and Systems Research, University of Birmingham, Edgbaston, B15 2TT, UK; a.b.sinclair@bham.ac.uk

Study funded by:

A.S. is funded by an NIHR Clinician Scientist Fellowship (NIHR-CS-011-028) and by the Medical Research Council, UK (MR/K015184/1).

Acknowledgments:

This work has been presented at the European Headache and Migraine Trust International Congress (Glasgow 2016); the UK CSF Disorders Meeting (Birmingham 2016) and the Keele Academic Trainee Conference (Keele 2017). With thanks to Peter Nightingale (Senior Statistician, University of Birmingham), for his advice on question framing for quantitative analysis of responses, as well as in planning and interpretation of statistical analysis.

Competing interests statement

None Declared.

Author's contribution to the research

William J. Scotton: Compilation of the survey results; statistical analysis; interpretation of the results and drafting the manuscript

Susan P. Mollan: Conception and design of the survey; critical review of the manuscript

Thomas Walters: Compilation of the survey results and statistical analysis

Sandra Doughty: Conception and design of the survey

Hannah Botfield Statistical analysis and interpretation of the results

Keira Markey: Interpretation off the results and drafting of the manuscript

Andreas Yiangou: Interpretation off the results and drafting of the manuscript

Shelly Williamson: Conception and design of the survey

Alexandra J. Sinclair: Conception and design of the survey; interpretation of the results and critical review of the manuscript

All authors have read and approved the final manuscript.

Data sharing statement: We are happy to share all data, including raw data if requested

For peer review only

For peer review only - http://bmjopen.bmj.com/site/about/guidelines.xhtml

Abstract

Objectives

Patients with idiopathic intracranial hypertension (IIH) usually require multiple lumbar punctures during the course of their disease, and often report significant morbidity associated with the procedure. The aim of this study was to assess the patient's experience of diagnostic lumbar puncture (LP) in IIH.

Design, methods and participants

A cross-sectional study of IIH patients was conducted using an online survey, with the questions designed in collaboration with IIH:UK (the UK IIH charity). Responses were collated over a two-month period from April to May 2015.

Results

502 patients responded to the survey, of which 463 were analysed for this study. 40% of patients described severe pain during the LP (VRS ≥8), and the median pain score during the LP was 7 (VRS, IQR 5-7). The majority of patients felt they received insufficient pain relief (85%). Levels of anxiety about future LP's were high (median VRS 7, IQR 4-10), with 47% being extremely anxious (VRS ≥8). LPs performed as an emergency were associated with significantly greater pain scores compared to elective procedures (median 7, IQR 5-7 vs. 6, IQR 4-8, p=0.012). Higher LP pain scores (VRS) were significantly associated with poorly informed patients (Spearman correlation, r=-0.32, p<0.001). Patients felt more informed when the LP was performed by Specialist Registrar compared to a Junior Doctor (median 7 vs. 5, p=0.001) or Consultant compared to Junior Doctor (median 8 vs. 5, p<0.001).

For peer review only

Conclusions

This study was commissioned by the IIH patient group and is the first to document the patient experience of diagnostic lumbar punctures in IIH. It shows that the majority of these patients are experiencing significant morbidity from pain and anxiety. Additionally, patient experience of LP may be improved through enhanced pre-procedural information, and where possible, avoiding emergency LP's in favour of LP's booked on an elective day case unit.

Strengths and limitations of this study

- This large sample size UK survey is the first known to directly and specifically document the patient experience of diagnostic lumbar punctures in IIH, and confirms that a significant number of these patients are experiencing morbidity from pain and anxiety related to the procedure.
- The use of an online questionnaire ensured anonymity, thus increasing the likelihood of honest reporting by patients of their subjective experiences of the procedure.
- Given the retrospective nature of this study, the results may be susceptible to recall bias, thus limiting the generalisability of our findings.
- The study suggests practical recommendations for areas in which we scan intervene to improve the patients' experience of diagnostic LP in IIH.

For peer review only

Introduction

Idiopathic intracranial hypertension (IIH) is characterised by raised intracranial pressure (ICP) which can cause papilloedema with significant visual loss in some, as well as severe disabling headaches which significantly impact on quality of life in the majority (1)(2). The diagnostic criteria for IIH is based on an elevated lumbar puncture (LP) opening pressure (≥250 mm CSF in adults) in a properly performed lumbar puncture (3).

Many patients have multiple LPs during the disease course typically to assess disease severity, and in some cases as a therapeutic strategy. Established complications of LPs include local discomfort, low pressure headaches and more rarely infection or local haemorrhage (4). We have been made aware of an additional significant complication of LPs voiced by the patients themselves. The patients describe a very negative experience of LP's associated with anxiety, fear and pain during and after the procedure. The National charity IIH:UK (Registered Charity in England and Wales no 1143522 & Scotland SCO43294) approached us with concerns about the IIH patient experience of LP's. Patient experience of spinal anaesthesia and LP has previously been studied (5)(6). However, the experience of IIH patients undergoing LP has not been evaluated. LP's are typically more technically challenging in the IIH population as over 90% of these patients are obese (3).

The aim of this study was to assess the patient's experience of diagnostic LPs in IIH. We aimed disseminate this evidence to medical professionals to increase awareness of this potential morbidity of LP in IIH patients. Furthermore, we aimed to use evidence from this study as a catalyst to drive improvements in patient care.

Material and Methods

The study was carried out in collaboration with IIH:UK who canvased their members through the charity's contact network to establish the initial interest in the subject area. IIH:UK established the question topics and contacted our clinical team to provide guidance

For peer review only

on how to frame quantitative questions. A cross-sectional study was then conducted using an online survey. IIH:UK sent a survey monkey questionnaire through social media outlets (Facebook, Twitter (@IIHUK) and IIH:UK charity website (www.IIHUK.org), and allowed a two-month period from 1st April to 31st May 2015 for responses. Questionnaires were excluded if the respondents were under the age of 16 years or the survey was incomplete (missing key data fields) or uninterpretable. Anonymised data was analysed by the clinical team with input from the clinical research facility statistician (PN).

The questionnaire (see supplementary document) detailed baseline demographic details (age, weight and height), and details of the LP (emergency versus planned procedure, hospital setting, number of attempts, whether went on to have an X-Ray guided procedure and seniority of doctor performing). Data on anxiety (for the LP and future LP's), pain experienced and extent of understanding of the procedure was also collected. Patients were asked to quantify responses using a verbal rating score (VRS) 0-10 with 0 being the minimum and 10 the maximum score.

Statistical analysis

Statistical analysis was performed using SPSS versions 24 (SPSS Inc., Chicago, IL, USA) and GraphPad Prism 7 for Windows (GraphPad Software Inc., La Jolla, CA, USA). Assessment of data for normality was performed for each analysis. Normally distributed data was reported using mean and standard deviation (SD), and non-normally distributed data was reported using median and interquartile range (IQR). For all comparisons of continuous variables, a non-parametric test was used due to non-normality of data distribution. For comparison of two medians Mann-Whitney U tests were used, whilst for comparison of multiple medians Kruskall-Wallis H tests were used. Spearman's rank-order correlation was used to analyse the correlation between how informed the patients were and how much pain they felt. For comparison of categorical variables Chi square tests were used. Values were considered statistically significant when P values were less than 0.05.

For peer review only

Results

Demographics

There were 502 responders to the study, of which 463 were eligible for analysis. 18 responders did not complete the survey, 11 were under the age of 16 years, and 10 gave incomplete answers or ambiguous information that could not be objectively interpreted (Figure 1). The mean age was 33 years (SD 8.9), 98.5% were female (n=456), with a mean weight of 97.4kg (SD 22.3), and a mean Body Mass Index (BMI) of 36 kg/m^2 (SD 8.3). The median number of LPs undergone since diagnosis was 4 (inter-quartile range [IQR] 1-11), though 3.1% of patients (n=15) reported more than 50 LPs. When number of LPs was adjusted to reflect length of disease, the median number of LPs per year since diagnosis was 1.3 (IQR 0.3-3.6) (Table 1).

Pain, Anxiety & Analgesia

The majority of patients indicated they were extremely scared about the imminent LP (median VRS 8, IQR 6-10), with 60% indicating a VRS ≥8 in relation to how scared they felt (Figure 2A & 2B). 40% of patients described severe pain during the LP (VRS ≥8) with a median pain score of 7 (VRS, IQR 5-8) (Figure 2A & 2B). Additionally, the majority of patients felt they received insufficient pain relief (85%). Levels of anxiety about future LP's were high (median VRS 7, IQR 4-10), with 47% being extremely anxious (VRS ≥8) (Figure 2A & 2D). There was no relationship found between the pre-procedure anxiety levels and the subsequent recalled pain score of the LP.

Setting of LP and pre-procedural information

LPs were predominantly performed in the emergency setting (72%), as opposed to as an elective planned procedure on day-case unit. Importantly the LPs performed as an emergency were associated with significantly greater pain scores compared to elective procedures (VRS median 7, IQR 5-7 vs 6, IQR 4-8 respectively, p=0.012).

For peer review only

Only 37% of patients felt well informed about LP pre-procedure (VRS ≥8); 27% felt poorly informed (VRS 0-3), and 7% did not feel they were informed at all (VRS 0). Higher LP pain scores (VRS) were significantly associated with patients being poorly informed (Spearman correlation, r=-0.32, p<0.001) (Figure 3B). Patients felt better informed if they had an elective planned LP compared to an emergency procedure (median 7, IQR 5-10 versus median 6, IQR 5-10, p=0.011).

Difficulty of LP and need for X-ray guided procedure

47% of patients had 2 or more doctors attempt their LP (median 1, IQR 1-2) while 45% had greater than 3 attempts (number of times needle inserted) before success (median 1, IQR 1-4). 10.7% went on to have an X-Ray guided procedure due to failure of the initial LP, and the BMI was significantly higher in this group (mean kg/m^2 40.3 vs. 35.5, p=0.001) (Figure 3A). Compared to those that had normal LPs, the patients having X-Ray guided procedures felt less informed (VRS median 3 vs 6, p=0.002), suffered more pain (VRS median 8 vs 7, p=0.004), and were more anxious about future LPs (VRS median 9 vs 7, p=0.003).

Grade of Doctor performing LP

Patients felt more informed when the LP was performed by a Specialist Registrar (SpR) compared to a Junior doctor (median VRS 7 vs 5, p=0.001) or Consultant compared to a Junior doctor (median VRS 8 vs 5, p< 0.001), though there was no significant difference in the pain scores reported. They also suffered from less severe post-LP headaches (SpR vs Junior median VRS 7 vs 8, p<0.001, Consultant vs Junior median VRS 6.5 vs 8, p=0.003), and length of post-LP headache (SpR vs Junior median days 3 vs 6, p=0.02, Consultant vs Junior median days 4 vs 6, p=0.9).

Discussion

This is the first study, to the best of our knowledge, to document the patient experience of diagnostic lumbar punctures in IIH. It has shown that a number of patients are recalling

For peer review only

significant pain and anxiety. This morbidity is associated with inadequate pre-procedural information, the environment the LP is performed in (emergency setting being associated with increased pain), and the seniority of the doctor performing the LP.

Anaesthetists have long recognised the importance of the patient experience of spinal anaesthesia as an outcome measure and an indicator of quality of care (5) (6) (7). This is reflected in the high satisfaction levels patients report with the procedure (96-97%), which is in stark contrast to the feedback here. The differences between the anaesthetic population and the IIH patient group may be related to the procedure being technically challenging due to the patient's high BMI, the procedure happening as an emergency and in some having multiple LPs during the course of their IIH. It may also be due to anaesthetists having better technical skills due to performing the procedure more often, in addition to more closely supervised and rigorous training .

This was a large sample size study (463 responders) where patients could respond anonymously, thus increasing the likelihood of honest reporting of their subjective experiences of the procedure. This cohort reports the LP experience as negative with 40% of patients experiencing severe pain (VRS ≥8) during the procedure, 85% saying they did not receive adequate analgesia and 47% stating they were extremely anxious (VRS ≥8) about future LPs.

The majority of the group did not feel they received adequate pre-procedural information, with 63% not feeling well informed (VRS <8), and 7% saying that they were not informed at all (VRS =0). Patient who were less informed experienced more pain during the procedure. This highlights a key area where simple steps (see Box 1) could be made which may improve patient care.

Environment also had a bearing on the patient experience with 72% of LPs being performed in the emergency setting; this was associated with the patient feeling less informed, and reporting significantly higher pain scores, compared to an elective procedure on a day-case unit. Optimisation of the environment for the patient undergoing LP could therefore positively affect their outcome (see Box 1). The day case environment may provide

For peer review only

access to doctors adequately trained in performing LPs as well as a less time-pressurised environment. Time to reflect on the procedure, read pre-procedural information and give informed consent may help improve the overall experience. Although diverting IIH patients away from the emergency room would likely benefit the majority, it may not be practical for a minority of papilloedema cases where there is progressive or rapid loss of visual function, and the need for acute diagnosis.

The study also suggests that there is also scope for improving our technical skills in LP, as 85% of our cohort stated that they did not receive adequate analgesia, with 45% undergoing greater than 3 attempts (defined as the needle being fully withdrawn between attempts), and 47% having 2 or more doctors attempt the LP. When the LP was performed by a doctor more junior that registrar (30% of the time), the patients felt less informed, and reported more serve and longer lasting post-LP headaches. We acknowledge that the grade of doctor performing the LP may not be accurately recalled by the patient and maybe more of a reflection of the patient's confidence in the doctor. However, diverting LPs into the day-case setting would provide an opportunity where the junior doctors could be appropriately supervised and trained, which has been shown to increase their ability to perform the procedure (8).

In this cohort, 10.7% of patients reported having an x-ray guided procedure due to failure of the initial LP, with the BMI being significantly higher in this group (mean 40.3kg/m^2 vs. 35.5kg/m^2, p=0.001). This finding is in keeping with a recent study which showed a strong correlation between BMI and procedure failure, with half of the failed LP's occurring in patients with a BMI greater than 35kg/m^2 (9). The inability to palpate landmarks in obese patients is likely to be a significant driver of this correlation (10). The x-ray guided LP group felt less informed, reported more pain, and were more likely to feel anxious about future LPs; findings most likely due to the number of failed attempts before the x-ray guided procedure. The growing evidence base for use of ultrasound guidance, particularly in patients with a higher BMI and absence of landmarks (11), suggests that its use in the IIH patient cohort

For peer review only

may increase the success rate of the initial LP and decrease the number that require x-ray guided procedures (see Box 1).

For clinical care a positive experience of a diagnostic LP will positively impact on the patient's future engagement with healthcare services, whilst in IIH research LP experience affects recruitment to clinical trials (12); it is therefore critical that clinicians optimise patient care.

Limitations

The main limitation of the study is its retrospective nature and as such the results are likely susceptible to recall bias. Interpretation of some of the study questions is problematic: for example, for questions such as the number of attempts for a lumbar puncture and the seniority of the doctor performing the procedure, the respondents may not accurately know the answer.

Conclusion

There has been a growing consensus in recent years that if healthcare services are to better deliver patient-centred care then research needs to be more reflective of patients' needs and concerns (13)(14). This study was commissioned by the IIH patient group and is the first to document the patient experience of diagnostic lumbar punctures in IIH. It documents experiences of significant pain and anxiety associated with both inadequate pre-procedural information and the setting the LP is performed in. The study suggests a number of practical steps that may improve the patient experience of LP's.

Recommendations for improving patient experience of diagnostic LP in IIH

- Providing enhanced pre-procedural information.
- Where possible, diverting emergency department LPs to elective procedures on dedicated day case units.

For peer review only

- Simulation training for doctors and specialist nurses to develop appropriate technical (including ultrasound guidance) and human factor skills (such as communication, empathy and leadership) for performing LPs in a technically difficult patient cohort.
- Implementing widespread patient reported outcome measures for LP's to guide the need for service improvements and training needs.

Bibliography

1. Mollan SP, Ali F, Hassan-Smith G, et al. Evolving evidence in adult idiopathic intracranial hypertension: pathophysiology and management. J Neurol Neurosurg Psychiatry. 2016;87(9):982-92
2. Mulla Y, Markey KA, Woolley RL, et al. Headache determines quality of life in idiopathic intracranial hypertension. J Headache Pain. 2015;16:521
3. Markey KA, Mollan SP, Jensen RH, et al. Understanding idiopathic intracranial hypertension: mechanisms, management, and future directions. Lancet Neurol. 2016;15(1):78-91.
4. Wright BLC, Lai JTF, Sinclair AJ. Cerebrospinal fluid and lumbar puncture: A practical review. J Neurol. 2012;259(8):1530–45.
5. Rhee WJ, Chung CJ, Lim YH, et al. Factors in patient dissatisfaction and refusal regarding spinal anesthesia. Korean J Anesthesiol. 2010;59(4):260-4
6. Dharmalingam TK, Ahmad Zainuddin NA. Survey on maternal satisfaction in receiving spinal anaesthesia for caesarean section. Malaysian J Med Sci. 2013;20(3):51–4.
7. Wu CL, Naqibuddin M;, Fleisher LA. Measurement of patient satisfaction as an outcome of regional anesthesia and analgesia: a systematic review. Reg Anesth Pain Med. 2001;26(3):196-208.
8. Sweeney M, Al-Diwani A, Hadden R. Improving the safety and efficiency of outpatient lumbar puncture service. BMJ Qual Improv Rep. 2016;25:5(1).

For peer review only

For peer review only - http://bmjopen.bmj.com/site/about/guidelines.xhtml

9. Edwards C, Leira EC, Gonzalez-Alegre P. Residency training: a failed lumbar puncture is more about obesity than lack of ability. Neurology. 2015 Mar 10;84(10):e69-72.

10. Stiffler KA, Jwayyed S, Wilber ST, et al. The use of ultrasound to identify pertinent landmarks for lumbar puncture. Am J Emerg Med. 2007;25(3):331-4.

11. Soni NJ, Franco-Sadud R, Schnobrich D, et al. Ultrasound guidance for lumbar puncture. Neurol Clin Pract. 2016;6(4):358-368.

12. Ball AK, Howman A, Wheatley K, et al. A randomised controlled trial of treatment for idiopathic intracranial hypertension. J Neurol. 2011;258(5):874-81.

13. Chalmers I, Bracken MB, Djulbegovic B, et al. How to increase value and reduce waste when research priorities are set. Lancet. 2014;383(9912):156-65.

14. Entwistle V, Calnan M, Dieppe P. Consumer involvement in setting the health services research agenda: persistent questions of value. J Health Serv Res Policy. 2008;13(3):76–81

Competing interests statement

None Declared.

Author's contribution to the research

William J. Scotton Compilation of the survey results; statistical analysis; interpretation of the results and drafting the manuscript

Susan P. Mollan Conception and design of the survey; critical review of the manuscript

Thomas Walters Compilation of the survey results and statistical analysis

Sandra Doughty Conception and design of the survey

Peter Nightingale Statistical analysis

Hannah Botfield Statistical analysis and interpretation of the results

Keira Markey: Interpretation off the results and drafting of the manuscript

Andreas Yiangou: Interpretation off the results and drafting of the manuscript

For peer review only

Shelly Williamson Conception and design of the survey

Alexandra J. Sinclair Conception and design of the survey; interpretation of the results and critical review of the manuscript

All authors have read and approved the final manuscript.

Tables

Table 1: Baseline characteristics of eligible responders

Variable	No. (%) n=463
Age, mean, years (SD)	32.9 (8.9)
Female sex	456 (98.5%)
Weight, mean, kg (SD)	97.2 (22.5)
BMI, mean (SD)	36.0 (8.3)
LPs since diagnosis, median (IQR)	4 (1-11)
LPs per year since diagnosis, median (IQR)	1 (1-4)

For peer review only

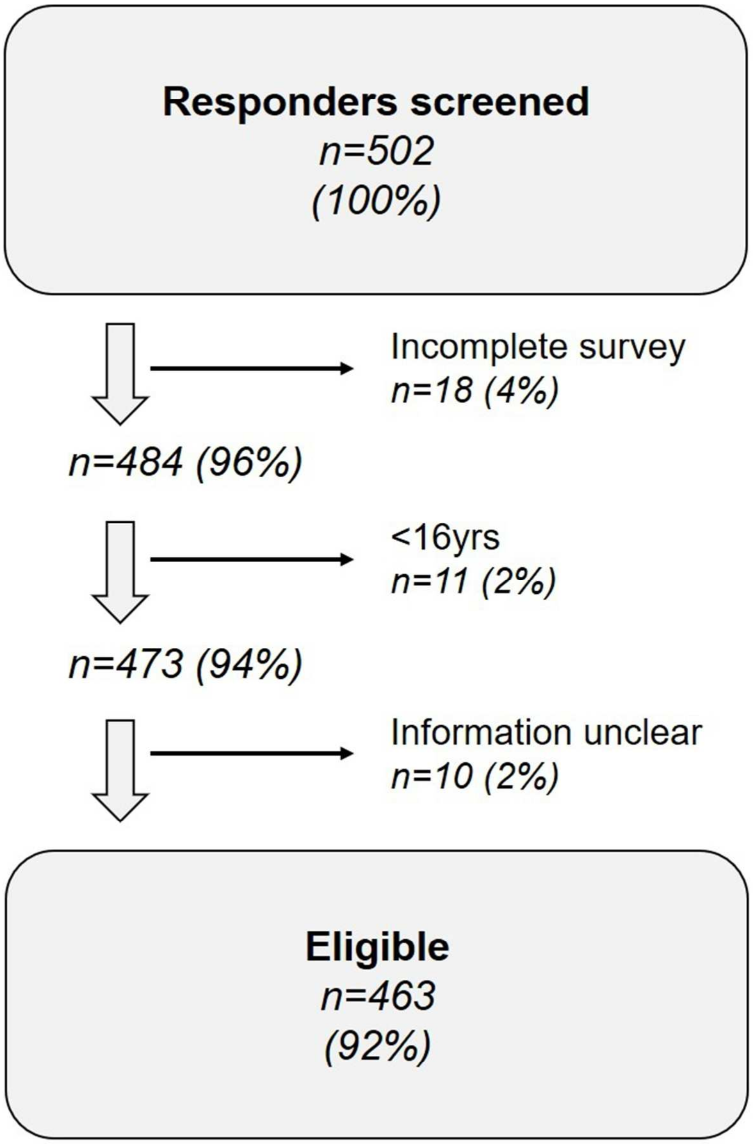

Table 1: Baseline characteristics of eligible responders

209x289mm (96 x 96 DPI)

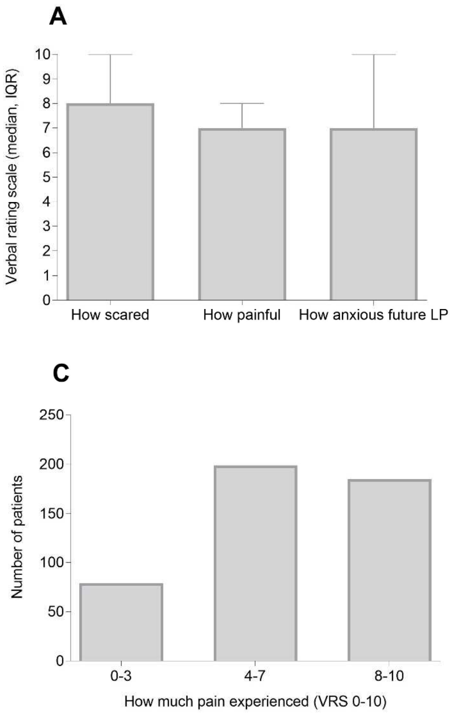

Figure 2: Patients' expectations and experience of LP. (A) Median VRS (0-10, IQR) for how scared patient was before LP, how painful the LP was and how anxious they were about future LPs. (B) Number of patients that were mildly (0-3), moderately (4-7), or very scared (8-10) before having an LP. (C) Number of patients that experienced mild (VRS 0-3), moderate (VRS 4-7) or severe (VRS 8-10) pain during the LP. (D) Number of patients that were mildly (VRS 0-3), moderately (VRS 4-7) or very anxious (VRS 8-10) about future LPs. [VRS 0=minimum and 10=maximal score]

284x212mm (300 x 300 DPI)

For peer review only - http://bmjopen.bmj.com/site/about/guidelines.xhtml

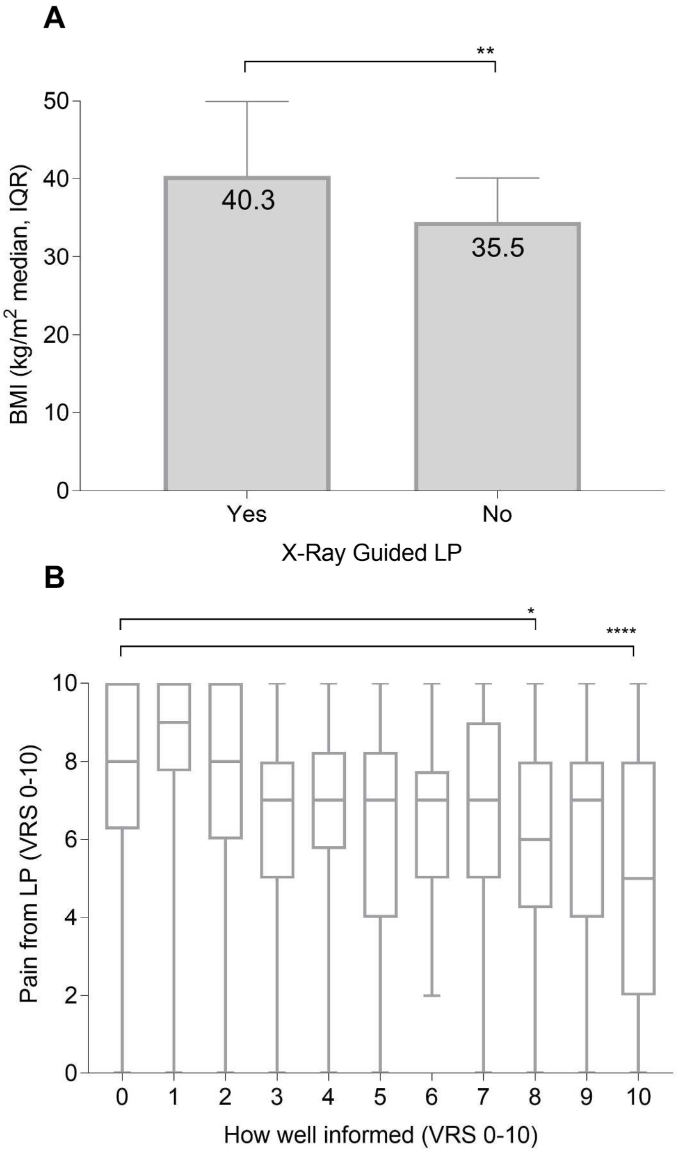

Figure 3: X-Ray guided LPs and relationship of pre-procedural information to patient experience. (A) BMI (median, IQR) and association with whether patient had X-Ray guided LP. (B) Association between how well-informed patient was before LP, and how painful LP was (median VRS, IQR, min-max). ns $p>0.05$, * $p \leq 0.05$, ** $p \leq 0.01$, *** $p \leq 0.001$. [VRS 0=minimum and 10=maximal score]

182x275mm (300 x 300 DPI)

IIHUK Online Questionnaire

Demographics

1. How old were you at time of diagnostic LP?
2. What was date of your first lumbar puncture? (Do not worry if you cannot remember the exact day, but please specify month and year)
3. Since your first diagnostic LP, how many LPs have you had? (Please approximate if not sure of the exact number)
4. What is your height (metres)?
5. What is your weight (kg)?

Details of lumbar puncture

1. Was your first admission regarding your LP planned (you were sent an appointment by your doctor) or as an emergency?
2. Where in the hospital did the first LP take place? (Emergency department, Ward or In Theatre)
3. How many different doctors attempted your diagnosing LP? (i.e how many different doctors tried inserting a needle)
4. How many attempts were made to get a diagnostic LP? (An attempt being defined as a needle being inserted)
5. If the initial LP failed to get a reading did you go on to have it done in theatre under X-ray guidance?

Patient experience of lumbar puncture

1. How frightened were you about the thought of the LP before the procedure on a scale 0-10? (0 being not frightened at all, 10 being the most scared you've ever felt)
2. How well do you feel you were informed about the procedure prior to the LP on a scale 0-10? (0 being not informed, 10 being fully informed)
3. Was adequate analgesia (i.e. local anaesthetic) used in your diagnosing LP?

For peer review only

4. How much pain did you experience during the LP on a scale of 0-10? (0 being pain free, 10 being worst pain ever experienced)
5. On a scale of 0-10, how anxious do you feel about having LPs in the future? (0 being not anxious, 10 being the most anxious you've ever felt)

For peer review only

STROBE Statement—checklist of items that should be included in reports of observational studies

	Item No	Recommendation
**Title and abstract**	1	(*a*) Indicate the study's design with a commonly used term in the title or the abstract (Pg1)
		(*b*) Provide in the abstract an informative and balanced summary of what was done and what was found (Pg 3-4)
**Introduction**		
Background/rationale	2	Explain the scientific background and rationale for the investigation being reported (Pg5)
Objectives	3	State specific objectives, including any prespecified hypotheses (Pg5)
**Methods**		
Study design	4	Present key elements of study design early in the paper (Pg5-6)
Setting	5	Describe the setting, locations, and relevant dates, including periods of recruitment, exposure, follow-up, and data collection (Pg6)
Participants	6	(*a*) *Cohort study*—Give the eligibility criteria, and the sources and methods of selection of participants. Describe methods of follow-up<br>*Case-control study*—Give the eligibility criteria, and the sources and methods of case ascertainment and control selection. Give the rationale for the choice of cases and controls<br>***Cross-sectional study***—Give the eligibility criteria, and the sources and methods of selection of participants (Pg6 & Figure 1)
		(*b*) *Cohort study*—For matched studies, give matching criteria and number of exposed and unexposed<br>*Case-control study*—For matched studies, give matching criteria and the number of controls per case
Variables	7	Clearly define all outcomes, exposures, predictors, potential confounders, and effect modifiers. Give diagnostic criteria, if applicable (Pg5-6)
Data sources/ measurement	8*	For each variable of interest, give sources of data and details of methods of assessment (measurement). Describe comparability of assessment methods if there is more than one group (Pg5-6)
Bias	9	Describe any efforts to address potential sources of bias (Pg11)
Study size	10	Explain how the study size was arrived at (Pg6)
Quantitative variables	11	Explain how quantitative variables were handled in the analyses. If applicable, describe which groupings were chosen and why (Pg 6)
Statistical methods	12	(*a*) Describe all statistical methods, including those used to control for confounding (Pg6)
		(*b*) Describe any methods used to examine subgroups and interactions (Pg6)
		(*c*) Explain how missing data were addressed (Pg6 – responders with missing data excluded from study)
		(*d*) *Cohort study*—If applicable, explain how loss to follow-up was addressed<br>*Case-control study*—If applicable, explain how matching of cases and controls was addressed<br>*Cross-sectional study*—If applicable, describe analytical methods taking account of sampling strategy (n/a)
		(*e*) Describe any sensitivity analyses (n/a)

For peer review only

Continued on next page

For peer review only

For peer review only - http://bmjopen.bmj.com/site/about/guidelines.xhtml

**Results**		
Participants	13*	(a) Report numbers of individuals at each stage of study—eg numbers potentially eligible, examined for eligibility, confirmed eligible, included in the study, completing follow-up, and analysed (Pg 7 & Figure 1)
		(b) Give reasons for non-participation at each stage (see above)
		(c) Consider use of a flow diagram (Figure 1)
Descriptive data	14*	(a) Give characteristics of study participants (eg demographic, clinical, social) and information on exposures and potential confounders (Pg 7 & Table 1)
		(b) Indicate number of participants with missing data for each variable of interest (Pg 7)
		(c) *Cohort study*—Summarise follow-up time (eg, average and total amount)
Outcome data	15*	*Cohort study*—Report numbers of outcome events or summary measures over time
		Case-control study—Report numbers in each exposure category, or summary measures of exposure
		Cross-sectional study—Report numbers of outcome events or summary measures (Pg7-8)
Main results	16	(*a*) Give unadjusted estimates and, if applicable, confounder-adjusted estimates and their precision (eg, 95% confidence interval). Make clear which confounders were adjusted for and why they were included (Pg7-8)
		(*b*) Report category boundaries when continuous variables were categorized (Pg7-8)
		(*c*) If relevant, consider translating estimates of relative risk into absolute risk for a meaningful time period (n/a)
Other analyses	17	Report other analyses done—eg analyses of subgroups and interactions, and sensitivity analyses (n/a)
**Discussion**		
Key results	18	Summarise key results with reference to study objectives (Pg 8-10)
Limitations	19	Discuss limitations of the study, taking into account sources of potential bias or imprecision. Discuss both direction and magnitude of any potential bias (Pg11)
Interpretation	20	Give a cautious overall interpretation of results considering objectives, limitations, multiplicity of analyses, results from similar studies, and other relevant evidence (Pg11)
Generalisability	21	Discuss the generalisability (external validity) of the study results (Pg9)
**Other information**		
Funding	22	Give the source of funding and the role of the funders for the present study and, if applicable, for the original study on which the present article is based (Pg2)

*Give information separately for cases and controls in case-control studies and, if applicable, for exposed and unexposed groups in cohort and cross-sectional studies.

Note: An Explanation and Elaboration article discusses each checklist item and gives methodological background and published examples of transparent reporting. The STROBE checklist is best used in conjunction with this article (freely available on the Web sites of PLoS Medicine at http://www.plosmedicine.org/, Annals of Internal Medicine at http://www.annals.org/, and Epidemiology at http://www.epidem.com/). Information on the STROBE Initiative is available at www.strobe-statement.org.

For peer review only

BMJ Open

Negative patient experience of lumbar puncture in Idiopathic Intracranial Hypertension: a cross-sectional online survey.

Journal:	*BMJ Open*
Manuscript ID	bmjopen-2017-020445.R1
Article Type:	Research
Date Submitted by the Author:	07-Mar-2018
Complete List of Authors:	Scotton, William; University of Birmingham, Institute of Metabolism and Systems Research; University Hospitals Birmingham NHS Foundation Trust, Department of Neurology<br>Mollan, Susan; Institute of Metabolism and Systems Research, College of Medical and Dental Sciences, University of Birmingham; Birmingham Neuro-Ophthalmology Unit, Ophthalmology Department, University Hospitals Birmingham NHS Trust, Queen Elizabeth Hospital Birmingham<br>Walters, Thomas; Institute of Metabolism and Systems Research, College of Medical and Dental Sciences, University of Birmingham<br>Doughty, Sandra; Idiopathic Intracranial Hypertension UK<br>Botfield, Hannah; Institute of Metabolism and Systems Research, College of Medical and Dental Sciences, University of Birmingham; Centre for Endocrinology, Diabetes and Metabolism, Birmingham Health Partners<br>Markey, Keira; Institute of Metabolism and Systems Research, College of Medical and Dental Sciences, University of Birmingham; University Hospitals Birmingham NHS Foundation Trust, Department of Neurology<br>Yiangou, Andreas; Institute of Metabolism and Systems Research, College of Medical and Dental Sciences, University of Birmingham; University Hospitals Birmingham NHS Foundation Trust, Department of Neurology<br>Williamson, Shelley; Idiopathic Intracranial Hypertension UK<br>Sinclair, AJ; Institute of Metabolism and Systems Research, College of Medical and Dental Sciences, University of Birmingham; Centre for Endocrinology, Diabetes and Metabolism, Birmingham Health Partners
**Primary Subject Heading**:	Neurology
Secondary Subject Heading:	Patient-centred medicine, Medical education and training, Ophthalmology, Evidence based practice
Keywords:	Adult neurology < NEUROLOGY, Neuro-ophthalmology < NEUROLOGY, NEUROPATHOLOGY, Neuro-ophthalmology < OPHTHALMOLOGY

SCHOLARONE™
Manuscripts

Title: Negative patient experience of lumbar puncture in Idiopathic Intracranial Hypertension: a cross-sectional online survey.

Authors: William J. Scotton[1,2], Susan P. Mollan[1,3], Thomas Walters[1], Sandra Doughty[4], Hannah Botfield[1,5], Keira Markey[1,2], Andreas Yiangou[1,2], *Shelly Williamson[4], *Alexandra J. Sinclair[1,5].

Institutions

1. Institute of Metabolism and Systems Research, College of Medical and Dental Sciences, University of Birmingham, Birmingham, B15 2TT, United Kingdom
2. Department of Neurology, University Hospitals Birmingham NHS Foundation Trust, B15 2TH, United Kingdom.
3. Birmingham Neuro-Ophthalmology Unit, Ophthalmology Department, University Hospitals Birmingham NHS Trust, Queen Elizabeth Hospital Birmingham, Birmingham, UK
4. Idiopathic Intracranial Hypertension UK, Registered Charity in England & Wales No. 1143522 & Scotland SC043294
5. Centre for Endocrinology, Diabetes and Metabolism, Birmingham Health Partners, B15 2TH, United Kingdom

*share joint senior author

For peer review only

Word Count Abstract: 355

Word Count Paper: 2567 (not including 67 recommendations, 111 strengths/weaknesses)

Table(s): 2

Figure(s): 3

Corresponding author:

Alexandra Sinclair, Institute of Metabolism and Systems Research, University of Birmingham, Edgbaston, B15 2TT, UK; a.b.sinclair@bham.ac.uk

For peer review only - http://bmjopen.bmj.com/site/about/guidelines.xhtml

Study funded by:

A.S. is funded by an NIHR Clinician Scientist Fellowship (NIHR-CS-011-028) and by the Medical Research Council, UK (MR/K015184/1).

Acknowledgments:

With thanks to Peter Nightingale (Senior Statistician, University of Birmingham), for his advice on question framing for quantitative analysis of responses, as well as in planning and interpretation of statistical analysis, and to the IIH UK as well as the IIH patient group without which this study would not have been possible

Competing interests statement

None Declared.

Author's contribution to the research

William J. Scotton: Compilation of the survey results; statistical analysis; interpretation of the results and drafting the manuscript

Susan P. Mollan: Conception and design of the survey; critical review of the manuscript

Thomas Walters: Compilation of the survey results and statistical analysis

Sandra Doughty: Conception and design of the survey

Hannah Botfield Statistical analysis and interpretation of the results

Keira Markey: Interpretation off the results and drafting of the manuscript

Andreas Yiangou: Interpretation off the results and drafting of the manuscript

Shelly Williamson: Conception and design of the survey

Alexandra J. Sinclair: Conception and design of the survey; interpretation of the results and critical review of the manuscript

All authors have read and approved the final manuscript.

Data sharing statement: We are happy to share all data, including raw data if requested

For peer review only

Abstract

Objectives

Patients with idiopathic intracranial hypertension (IIH) usually require multiple lumbar punctures during the course of their disease, and often report significant morbidity associated with the procedure. The aim of this study was to assess the patient's experience of diagnostic lumbar puncture (LP) in IIH.

Design, methods and participants

A cross-sectional study of IIH patients was conducted using an anonymous online survey, with the questions designed in collaboration with IIH UK (the UK IIH charity). Responses were collated over a two-month period from April to May 2015. Patients were asked to quantify responses using a verbal rating score (VRS) 0-10 with 0 being the minimum and 10 the maximum score.

Results

502 patients responded to the survey, of which 463 were analysed for this study. 40% of patients described severe pain during the LP (VRS ≥8), and the median pain score during the LP was 7 (VRS, IQR 5-7). The majority of patients felt they received insufficient pain relief (85%). Levels of anxiety about future LPs were high (median VRS 7, IQR 4-10), with 47% being extremely anxious (VRS ≥8). LPs performed as an emergency were associated with significantly greater pain scores compared to elective procedures (median 7, IQR 5-7 vs. 6, IQR 4-8, p=0.012). 10.7% went on to have an X-Ray guided procedure due to failure of the initial LP, and the BMI was significantly higher in this group (mean kg/m^2 40.3 vs. 35.5, p=0.001). Higher LP pain scores (VRS) were significantly associated with poorly informed patients (Spearman correlation, r=-0.32, p<0.001). Patients felt more informed when the LP was performed by Specialist Registrar compared to a Junior Doctor (median 7 vs. 5, p=0.001) or Consultant compared to Junior Doctor (median 8 vs. 5, p<0.001).

For peer review only

Conclusions

This study was commissioned by the IIH patient group and is the first to document the patient experience of diagnostic lumbar punctures in IIH. It shows that the majority of these patients are experiencing significant morbidity from pain and anxiety. Patient experience of LP may be improved through changing clinical practice to include universal detailed pre-procedural information, and where possible, avoiding emergency LPs in favour of LP's booked on an elective day case unit (Box 1).

Box 1: Strengths and limitations of this study

- This large sample size UK survey is the first known to directly and specifically document the patient experience of diagnostic lumbar punctures in IIH, and confirms that a significant number of these patients are experiencing morbidity from pain and anxiety related to the procedure.
- The use of an online questionnaire ensured anonymity, thus increasing the likelihood of honest reporting by patients of their subjective experiences of the procedure.
- Given the retrospective nature of this study, the results may be susceptible to recall bias, thus limiting the generalisability of our findings.
- The study suggests practical recommendations for areas in which we can intervene to improve the patients' experience of diagnostic LP in IIH.

For peer review only

Introduction

Idiopathic intracranial hypertension (IIH) is characterised by raised intracranial pressure (ICP) which can cause papilloedema with significant visual loss in some, as well as severe disabling headaches which significantly impact on quality of life in the majority (1)(2). The diagnostic criteria for IIH is based on an elevated lumbar puncture (LP) opening pressure (≥250 mm CSF in adults) in a properly performed lumbar puncture (3).

Many patients have multiple LPs during the disease course typically to assess disease severity, and in some cases as a therapeutic strategy. Established complications of LPs include local discomfort, low pressure headaches and more rarely infection or local haemorrhage (4). We have been made aware of an additional significant complication of LPs voiced by the patients themselves. The patients describe a very negative experience of LP's associated with anxiety, fear and pain during and after the procedure. The National charity IIH:UK (Registered Charity in England and Wales no 1143522 & Scotland SCO43294) approached us with concerns about the IIH patient experience of LP's. Patient experience of spinal anaesthesia and LP has previously been studied (5)(6). However, the experience of IIH patients undergoing LP has not been evaluated. LP's are typically more technically challenging in the IIH population as over 90% of these patients are obese (3).

The aim of this study was to assess the patient's experience of diagnostic LPs in IIH. We aimed disseminate this evidence to medical professionals to increase awareness of this potential morbidity of LP in IIH patients. Furthermore, we aimed to use evidence from this study as a catalyst to drive improvements in patient care.

Material and Methods

Public and Patient Involvement

This research was initiated, designed and conducted by IIH UK, a charity who supports IIH patients and carers. The charity agreed at a Trustee meeting to design a survey to investigate the magnitude of lumbar puncture related anxiety in response to their overwhelming messages from patients. When the first survey was performed, the Trustees

For peer review only

recognised that they would need help in analysis of the data and ask addition questions so a further survey was conducted. The clinical researchers at the University Hospitals Birmingham provided support with statistical analysis and critical review of the data.

Dissemination of the results was planned via physician and patient meetings, through medical and patient lead social media, and on the IIH UK patients' charity website.

Study Design

The cross-sectional study was conducted using an online survey. IIH UK sent a survey monkey questionnaire through social media outlets (Facebook, Twitter (@IIHUK) and IIH UK charity website (www.IIHUK.org), and allowed a two-month period from 1st April to 31st May 2015 for responses. Questionnaires were excluded if the respondents were under the age of 16 years or the survey was incomplete (missing key data fields) or uninterpretable. Anonymised data was analysed by the clinical team with input from the clinical research facility statistician (PN). As the charity board had already agreed with their members beforehand, and both surveys instructed the respondents that the information would be used to be published within the medical literature, no further ethical approval was required.

The questionnaire (see supplementary document) detailed baseline demographic details (age, weight and height), and details of the LP (emergency versus planned procedure, hospital setting, number of attempts, whether went on to have an X-Ray guided procedure and seniority of doctor performing). Data on anxiety (for the LP and future LP's), pain experienced and extent of understanding of the procedure was also collected. Patients were asked to quantify responses using a verbal rating score (VRS) 0-10 with 0 being the minimum and 10 the maximum score.

Statistical analysis

Statistical analysis was performed using SPSS versions 24 (SPSS Inc., Chicago, IL, USA) and GraphPad Prism 7 for Windows (GraphPad Software Inc., La Jolla, CA, USA). Assessment of data for normality was performed for each analysis. Normally distributed data was reported using mean and standard deviation (SD), and non-normally distributed data

For peer review only

was reported using median and interquartile range (IQR). For all comparisons of continuous variables, a non-parametric test was used due to non-normality of data distribution. For comparison of two medians Mann-Whitney U tests were used, whilst for comparison of multiple medians Kruskall-Wallis H tests were used. Spearman's rank-order correlation was used to analyse the correlation between how informed the patients were and how much pain they felt, as well as between BMI and how scared a patient was beforehand, how much pain they felt, and how anxious they felt about future LPs. For comparison of categorical variables Chi square tests were used. Values were considered statistically significant when P values were less than 0.05.

Results

Demographics

There were 502 responders to the study, of which 463 were eligible for analysis. 18 responders did not complete the survey, 11 were under the age of 16 years, and 10 gave incomplete answers or ambiguous information that could not be objectively interpreted (Figure 1). The mean age was 33 years (SD 8.9), 98.5% were female (n=456), with a mean weight of 97.4kg (SD 22.3), and a mean Body Mass Index (BMI) of 36 kg/m^2 (SD 8.3). The median number of LPs undergone since diagnosis was 4 (inter-quartile range [IQR] 1-11), though 3.1% of patients (n=15) reported more than 50 LPs. When number of LPs was adjusted to reflect length of disease, the median number of LPs per year since diagnosis was 1.3 (IQR 0.3-3.6) (Table 1).

Pain, Anxiety & Analgesia

The majority of patients indicated they were extremely scared about the imminent LP (median VRS 8, IQR 6-10), with 60% indicating a VRS ≥8 in relation to how scared they felt (Figure 2A & 2B). 40% of patients described severe pain during the LP (VRS ≥8) with a median pain score of 7 (VRS, IQR 5-8) (Figure 2A & 2C). Additionally, the majority of patients felt they received insufficient pain relief (85%). Levels of anxiety about future LP's

For peer review only

were high (median VRS 7, IQR 4-10), with 47% being extremely anxious (VRS ≥8) (Figure 2A & 2D). There was no relationship found between the pre-procedure anxiety levels and the subsequent recalled pain score of the LP.

Setting of LP and pre-procedural information

LPs were predominantly performed in the emergency setting (72%), as opposed to as an elective planned procedure on day-case unit. Importantly the LPs performed in the emergency setting were associated with significantly greater pain scores compared to elective procedures (VRS median 7, IQR 5-7 vs 6, IQR 4-8 respectively, p=0.012).

Only 37% of patients felt well informed about LP pre-procedure (VRS ≥8); 27% felt poorly informed (VRS 0-3), and 7% did not feel they were informed at all (VRS 0). Higher LP pain scores (VRS) were significantly associated with patients being poorly informed (Spearman correlation, r=-0.32, p<0.001) (Figure 3A). Patients felt better informed if they had an elective planned LP compared to a procedure in the emergency setting(median 7, IQR 5-10 versus median 6, IQR 5-10, p=0.011).

Difficulty of LP and need for X-ray guided procedure

47% of patients had 2 or more doctors attempt their LP (median 1, IQR 1-2) while 45% had greater than 3 attempts (number of times needle inserted) before success. 10.7% went on to have an X-Ray guided procedure due to failure of the initial LP, and the BMI was significantly higher in this group (mean kg/m^2 40.3 vs. 35.5, p=0.001) (Figure 3B). There was only a weak correlation between BMI and how scared a patient was beforehand, how much pain they felt, and how anxious they felt about future LPs (Spearman r = 0.17, 0.17, 0.17 respectively, p<0.001 for all). Compared to those that had normal LPs, the patients having X-Ray guided procedures felt less informed (VRS median 3 vs 6, p=0.002), suffered more pain (VRS median 8 vs 7, p=0.004), and were more anxious about future LPs (VRS median 9 vs 7, p=0.003).

For peer review only

Grade of Doctor performing LP

Table 2 shows the number of LP attempts by grade of doctor performing the LP. Patients felt more informed when the LP was performed by a Specialist Registrar (SpR) compared to a Junior doctor (median VRS 7 vs 5, p=0.001) or Consultant compared to a Junior doctor (median VRS 8 vs 5, p< 0.001), though there was no significant difference in the pain scores reported. They also suffered from less severe post-LP headaches (SpR vs Junior median VRS 7 vs 8, p<0.001, Consultant vs Junior median VRS 6.5 vs 8, p=0.003) (Figure 3C), and length of post-LP headache (SpR vs Junior median days 3 vs 6, p=0.02, Consultant vs Junior median days 4 vs 6, p=0.9) (Figure 3D).

Discussion

This is the first study, to the best of our knowledge, to document the patient experience of diagnostic lumbar punctures in IIH. It has shown that a number of patients are recalling significant pain and anxiety. This morbidity is associated with inadequate pre-procedural information, the environment the LP is performed in (emergency setting being associated with increased pain), and the seniority of the doctor performing the LP.

Anaesthetists have long recognised the importance of the patient experience of spinal anaesthesia as an outcome measure and an indicator of quality of care (5) (6) (7). This is reflected in the high satisfaction levels patients report with the procedure (96-97%), which is in stark contrast to the feedback here. The differences between the anaesthetic population and the IIH patient group may be related to the procedure being technically more challenging due to the patient's high BMI, the procedure happening as an emergency and some having multiple LPs during the course of their IIH. It may also be due to anaesthetists having better technical skills due to performing the procedure more often than the doctors (often non-neurologists) performing the LPs in the emergency setting, in addition to more closely supervised and rigorous training .

This was a large sample size study (463 responders) where patients could respond anonymously, thus increasing the likelihood of honest reporting of their subjective

For peer review only

experiences of the procedure. This cohort reports the LP experience as negative with 40% of patients experiencing severe pain (VRS ≥8) during the procedure, 85% saying they did not receive adequate analgesia and 47% stating they were extremely anxious (VRS ≥8) about future LPs.

The majority of the group did not feel they received adequate pre-procedural information, with 63% not feeling well informed (VRS <8), and 7% saying that they were not informed at all (VRS =0). Patients who were less informed experienced more pain during the procedure. Although all patients will have undergone a consent process in the UK, this data highlights the variable quality of the information disseminated by the physician to the patient. Current practices for informing patients about LP are likely to be highly variable across the UK. This study highlights a key area where simple changes in clinical practice to ensure all patients are provided with detailed pre-procedure information (Box 2) could facilitate improved patient care.

Environment also had a bearing on the patient experience with 72% of LPs being performed in the emergency setting; this was associated with the patient feeling less informed, and reporting significantly higher pain scores, compared to an elective procedure on a day-case unit. The high portion of the respondents who had an LP performed as part of an emergency admission in this study likely reflects the UK health care services and clinical practice where patients with a flare up in IIH symptoms are typically initially seen in the accident and emergency department for initial evaluation and often have a LP as part of their evaluation here, or on the acute medical unit. As the study was not designed to determine the clinical indications for the LP in each case no further inference can be made here. Typically, LP's performed in the emergency setting may be conducted by junior physicians, with less experience in conducting LP's than a speciality trained neurologist or anaesthetist. This may be a factor contributing to the poorer outcomes from LPs performed in the emergency setting.

Optimisation of the environment for the patient undergoing LP could therefore positively affect their outcome (see Box 1). The day case environment may provide access to doctors

For peer review only

adequately trained in performing LPs as well as a less time-pressurised environment. Time to reflect on the procedure and read pre-procedural information, may help improve the overall experience. Although diverting IIH patients away from the emergency setting would likely benefit the majority, it may not be practical for a minority of papilloedema cases where there is progressive or rapid loss of visual function, and the need for acute diagnosis.

The study also suggests that there is also scope for improving our technical skills in LP, as 85% of our cohort stated that they did not receive adequate analgesia, with 45% undergoing greater than 3 attempts (defined as the needle being fully withdrawn between attempts), and 47% having 2 or more doctors attempt the LP. When the LP was performed by a doctor more junior than registrar (30% of the time), the patients felt less informed, and reported more serve and longer lasting post-LP headaches. We acknowledge that the grade of doctor performing the LP may not be accurately recalled by the patient and maybe more of a reflection of the patient's confidence in the doctor. However, diverting LPs into the day-case setting would provide an opportunity where the junior doctors could be appropriately supervised and trained, which has been shown to increase their ability to perform the procedure (8).

In this cohort, 10.7% of patients reported having an x-ray guided procedure due to failure of the initial LP, with the BMI being significantly higher in this group (mean 40.3kg/m^2 vs. 35.5kg/m^2, p=0.001). This finding is in keeping with a recent study which showed a strong correlation between BMI and procedure failure, with half of the failed LPs occurring in patients with a BMI greater than 35kg/m^2 (9). The inability to palpate landmarks in obese patients is likely to be a significant driver of this correlation (10). The x-ray guided LP group felt less informed, reported more pain, and were more likely to feel anxious about future LPs; findings most likely due to the number of failed attempts before the x-ray guided procedure. The growing evidence base for use of ultrasound guidance, particularly in patients with a higher BMI and absence of landmarks (11), suggests that its use in the IIH patient cohort may increase the success rate of the initial LP and decrease the number that require x-ray guided procedures (see Box 1).

For peer review only

For clinical care a positive experience of a diagnostic LP will positively impact on the patient's future engagement with healthcare services, whilst in IIH research LP experience affects recruitment to clinical trials (12); it is therefore critical that clinicians optimise patient care.

Limitations

The main limitation of the study is its retrospective nature and as such the results are likely susceptible to recall bias. Interpretation of some of the study questions is problematic: for example, for questions such as the number of attempts for a lumbar puncture and the seniority of the doctor performing the procedure, the respondents may not accurately know the answer.

Conclusion

There has been a growing consensus in recent years that if healthcare services are to better deliver patient-centred care then research needs to be more reflective of patients' needs and concerns (13)(14). This study was commissioned by the IIH patient group and is the first to document the patient experience of diagnostic lumbar punctures in IIH. It documents experiences of significant pain and anxiety associated with both inadequate pre-procedural information and the setting the LP is performed in. The study suggests a number of practical steps that may improve the patient experience of LPs.

Box 2: Recommendations for improving patient experience of diagnostic LP in IIH

- Providing enhanced pre-procedural information.
- Where possible, diverting emergency department LPs to elective procedures on dedicated day case units.
- Simulation training for doctors and specialist nurses to develop appropriate technical (including ultrasound guidance) and human factor skills (such as

For peer review only

communication, empathy and leadership) for performing LPs in a technically difficult patient cohort.

- Implementing widespread patient reported outcome measures for LPs to guide the need for service improvements and training needs.

297

298 **Bibliography**

299 1. Mollan SP, Ali F, Hassan-Smith G, et al. Evolving evidence in adult idiopathic
300 intracranial hypertension: pathophysiology and management. J Neurol Neurosurg
301 Psychiatry. 2016;87(9):982-92
302 2. Mulla Y, Markey KA, Woolley RL, et al. Headache determines quality of life in
303 idiopathic intracranial hypertension. J Headache Pain. 2015;16:521
304 3. Markey KA, Mollan SP, Jensen RH, et al. Understanding idiopathic intracranial
305 hypertension: mechanisms, management, and future directions. Lancet Neurol.
306 2016;15(1):78-91.
307 4. Wright BLC, Lai JTF, Sinclair AJ. Cerebrospinal fluid and lumbar puncture: A practical
308 review. J Neurol. 2012;259(8):1530–45.
309 5. Rhee WJ, Chung CJ, Lim YH, et al. Factors in patient dissatisfaction and refusal
310 regarding spinal anesthesia. Korean J Anesthesiol. 2010;59(4):260-4
311 6. Dharmalingam TK, Ahmad Zainuddin NA. Survey on maternal satisfaction in receiving
312 spinal anaesthesia for caesarean section. Malaysian J Med Sci. 2013;20(3):51–4.
313 7. Wu CL, Naqibuddin M;, Fleisher LA. Measurement of patient satisfaction as an
314 outcome of regional anesthesia and analgesia: a systematic review. Reg Anesth Pain
315 Med. 2001;26(3):196-208.
316 8. Sweeney M, Al-Diwani A, Hadden R. Improving the safety and efficiency of outpatient
317 lumbar puncture service. BMJ Qual Improv Rep. 2016;25:5(1).
318 9. Edwards C, Leira EC, Gonzalez-Alegre P. Residency training: a failed lumbar
319 puncture is more about obesity than lack of ability. Neurology. 2015 Mar

For peer review only

10;84(10):e69-72.

10. Stiffler KA, Jwayyed S, Wilber ST, et al. The use of ultrasound to identify pertinent landmarks for lumbar puncture. Am J Emerg Med. 2007;25(3):331-4.
11. Soni NJ, Franco-Sadud R, Schnobrich D, et al. Ultrasound guidance for lumbar puncture. Neurol Clin Pract. 2016;6(4):358-368.
12. Ball AK, Howman A, Wheatley K, et al. A randomised controlled trial of treatment for idiopathic intracranial hypertension. J Neurol. 2011;258(5):874-81.
13. Chalmers I, Bracken MB, Djulbegovic B, et al. How to increase value and reduce waste when research priorities are set. Lancet. 2014;383(9912):156-65.
14. Entwistle V, Calnan M, Dieppe P. Consumer involvement in setting the health services research agenda: persistent questions of value. J Health Serv Res Policy. 2008;13(3):76–81

Competing interests statement

None Declared.

Author's contribution to the research

William J. Scotton Compilation of the survey results; statistical analysis; interpretation of the results and drafting the manuscript

Susan P. Mollan Conception and design of the survey; critical review of the manuscript

Thomas Walters Compilation of the survey results and statistical analysis

Sandra Doughty Conception and design of the survey

Peter Nightingale Statistical analysis

Hannah Botfield Statistical analysis and interpretation of the results

Keira Markey: Interpretation off the results and drafting of the manuscript

Andreas Yiangou: Interpretation off the results and drafting of the manuscript

Shelly Williamson Conception and design of the survey

For peer review only

Alexandra J. Sinclair Conception and design of the survey; interpretation of the results and critical review of the manuscript

All authors have read and approved the final manuscript.

Figure 1: Baseline characteristics of eligible responders

Table 1: Baseline characteristics of eligible responders

Variable	No. (%) n=463
Age, mean, years (SD)	32.9 (8.9)
Female sex	456 (98.5%)
Weight, mean, kg (SD)	97.2 (22.5)
BMI, mean (SD)	36.0 (8.3)
LPs since diagnosis, median (IQR)	4 (1-11)
LPs per year since diagnosis, median (IQR)	1 (1-4)

Figure 2: Patients' expectations and experience of LP. (A) Median VRS (0-10, IQR) for how scared patient was before LP, how painful the LP was and how anxious they were about future LPs. (B) Number of patients that were mildly (0-3), moderately (4-7), or very scared (8-10) before having an LP. (C) Number of patients that experienced mild (VRS 0-3), moderate (VRS 4-7) or severe (VRS 8-10) pain during the LP. (D) Number of patients that were mildly (VRS 0-3), moderately (VRS 4-7) or very anxious (VRS 8-10) about future LPs. [VRS 0=minimum and 10 maximal score]

For peer review only

Figure 3: X-Ray guided LPs, and relationship of pre-procedural information and grade of doctor performing LP to patient experience. (A) For all patients surveyed, association between how well-informed patient was before LP, and how painful LP was (median VRS, IQR, min-max). (B) BMI (median, IQR) and association with whether patient had X-Ray guided LP. (C) Grade of doctor performing LP and duration of post-LP headache (days, median, IQR). (D) Grade of doctor performing LP and severity of post-LP headache. ns $p>0.05$, * $p \leq 0.05$, ** $p \leq 0.01$, *** $p \leq 0.001$, [VRS 0=minimum and 10=maximal score].

Table 2: Number of LP attempts by grade of doctor

**Number of LP attempts**	**Grade of Doctor (% of total patients [n=463])**				
	Unknown	Junior	Registrar	Consultant	**Total**
1-3	13.0%	9.1%	15.1%	11.0%	**48.2%**
4-6	4.8%	5.2%	6.3%	3.9%	**20.1%**
7-9	2.6%	1.9%	3.0%	1.7%	**9.3%**
10-14	0.6%	1.7%	1.3%	0.4%	**4.1%**
15-19	0.4%	1.1%	0.4%	0.0%	**1.9%**
20+	0.6%	1.1%	0.4%	0.2%	**2.4%**
Unknown	4.5%	1.1%	3.2%	5.2%	**14.0%**
**Total**	**26.6%**	**21.2%**	**29.8%**	**22.5%**	**100%**

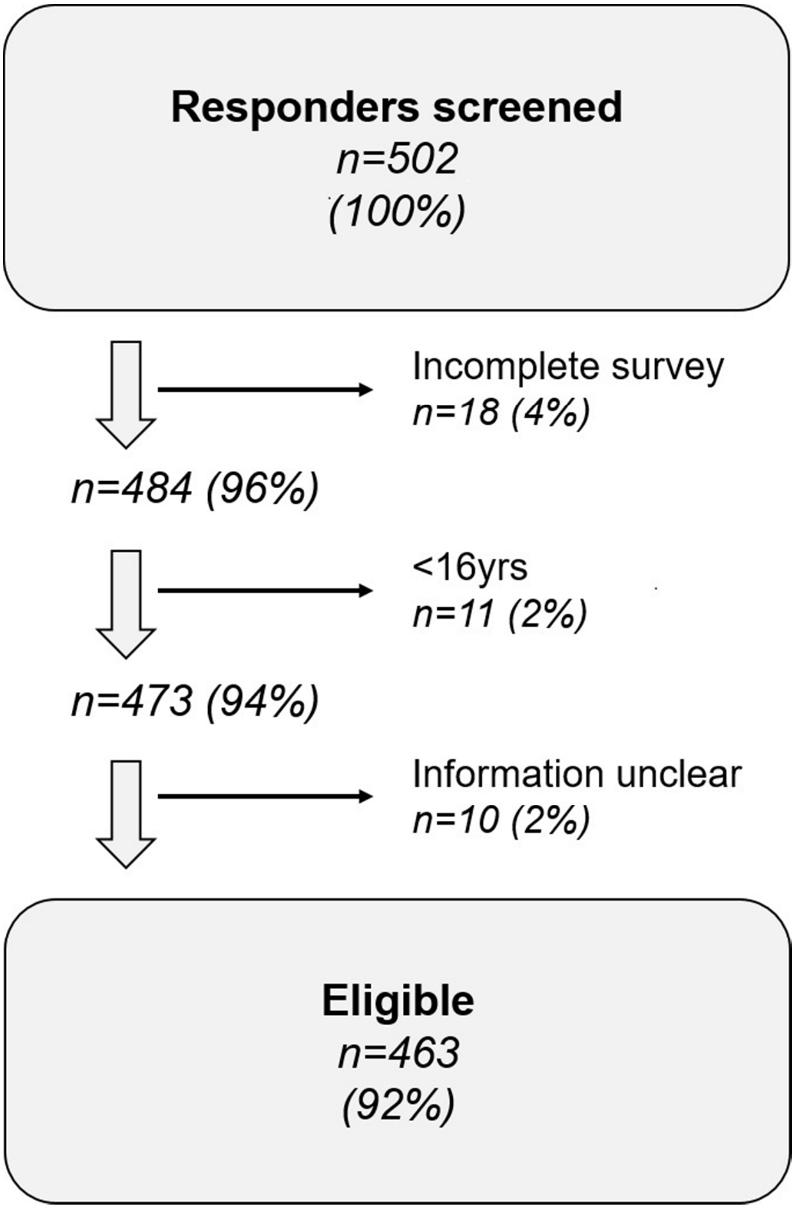

Figure 1: Baseline characteristics of eligible responders.

57x87mm (300 x 300 DPI)

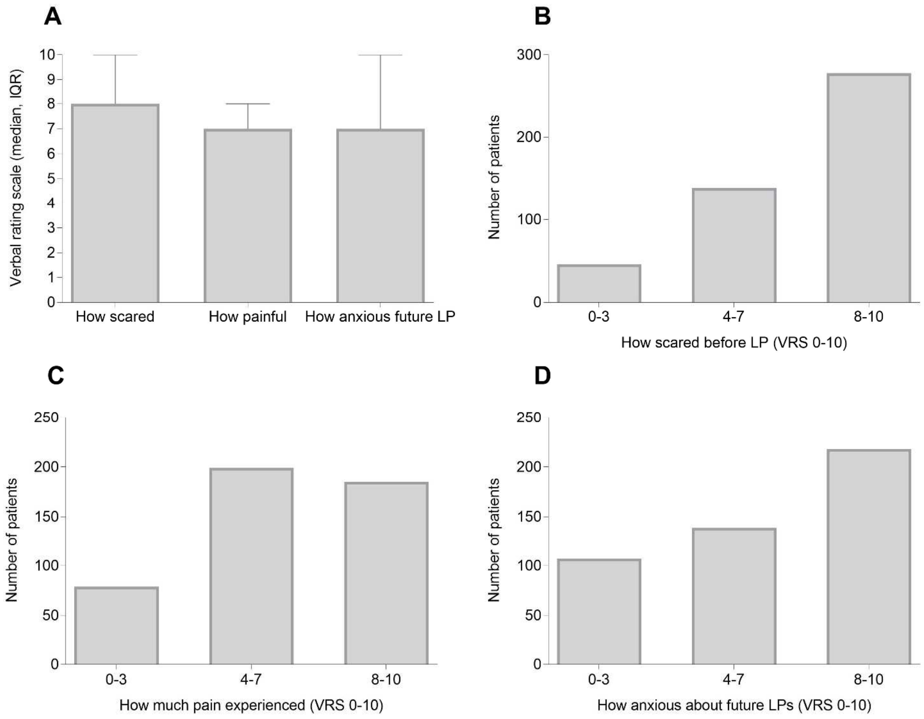

Figure 2: Patients' expectations and experience of LP. (A) Median VRS (0-10, IQR) for how scared patient was before LP, how painful the LP was and how anxious they were about future LPs. (B) Number of patients that were mildly (0-3), moderately (4-7), or very scared (8-10) before having an LP. (C) Number of patients that experienced mild (VRS 0-3), moderate (VRS 4-7) or severe (VRS 8-10) pain during the LP. (D) Number of patients that were mildly (VRS 0-3), moderately (VRS 4-7) or very anxious (VRS 8-10) about future LPs. [VRS 0=minimum and 10 maximal score].

282x212mm (300 x 300 DPI)

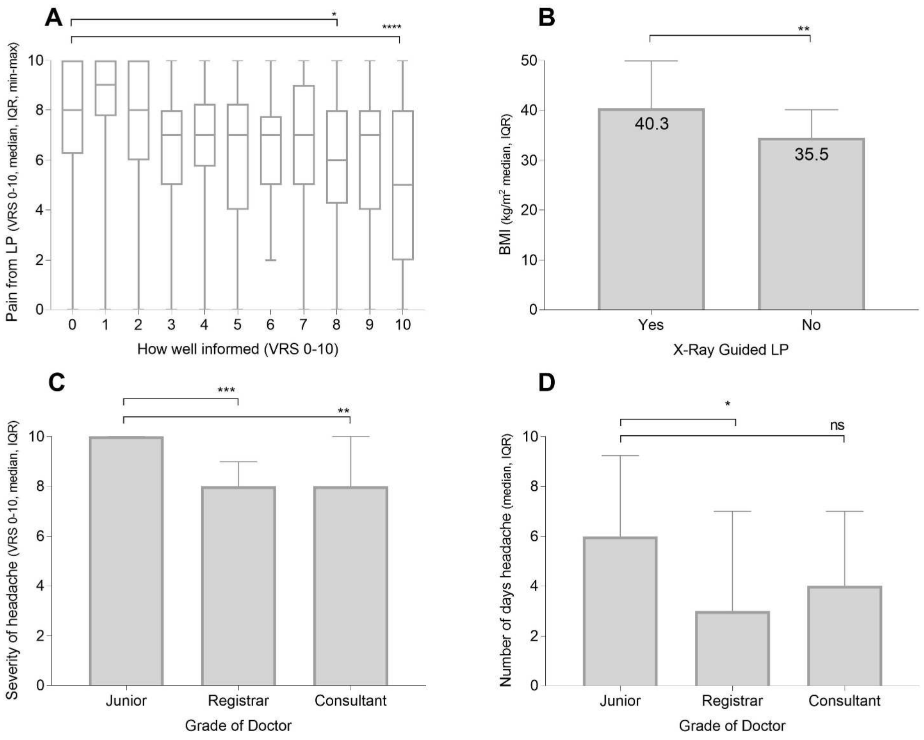

Figure 3: X-Ray guided LPs, and relationship of pre-procedural information and grade of doctor performing LP to patient experience. (A) For all patients surveyed, association between how well-informed patient was before LP, and how painful LP was (median VRS, IQR, min-max). (B) BMI (median, IQR) and association with whether patient had X-Ray guided LP. (C) Grade of doctor performing LP and duration of post-LP headache (days, median, IQR). (D) Grade of doctor performing LP and severity of post-LP headache. ns $p>0.05$, * $p \leq 0.05$, ** $p \leq 0.01$, *** $p \leq 0.001$, [VRS 0=minimum and 10=maximal score].

280x217mm (300 x 300 DPI)

IIHUK Online Questionnaire

Demographics

1. How old were you at time of diagnostic LP?
2. What was date of your first lumbar puncture? (Do not worry if you cannot remember the exact day, but please specify month and year)
3. Since your first diagnostic LP, how many LPs have you had? (Please approximate if not sure of the exact number)
4. What is your height (metres)?
5. What is your weight (kg)?

Details of lumbar puncture

1. Was your first admission regarding your LP planned (you were sent an appointment by your doctor) or as an emergency?
2. Where in the hospital did the first LP take place? (Emergency department, Ward or In Theatre)
3. How many different doctors attempted your diagnosing LP? (i.e how many different doctors tried inserting a needle)
4. How many attempts were made to get a diagnostic LP? (An attempt being defined as a needle being inserted)
5. If the initial LP failed to get a reading did you go on to have it done in theatre under X-ray guidance?

Patient experience of lumbar puncture

1. How frightened were you about the thought of the LP before the procedure on a scale 0-10? (0 being not frightened at all, 10 being the most scared you've ever felt)
2. How well do you feel you were informed about the procedure prior to the LP on a scale 0-10? (0 being not informed, 10 being fully informed)
3. Was adequate analgesia (i.e. local anaesthetic) used in your diagnosing LP?

For peer review only

4. How much pain did you experience during the LP on a scale of 0-10? (0 being pain free, 10 being worst pain ever experienced)
5. On a scale of 0-10, how anxious do you feel about having LPs in the future? (0 being not anxious, 10 being the most anxious you've ever felt)

For peer review only

STROBE Statement—checklist of items that should be included in reports of observational studies

	Item No	Recommendation
**Title and abstract**	1	(*a*) Indicate the study's design with a commonly used term in the title or the abstract (Pg1)
		(*b*) Provide in the abstract an informative and balanced summary of what was done and what was found (Pg 3-4)
**Introduction**		
Background/rationale	2	Explain the scientific background and rationale for the investigation being reported (Pg5)
Objectives	3	State specific objectives, including any prespecified hypotheses (Pg5)
**Methods**		
Study design	4	Present key elements of study design early in the paper (Pg5-6)
Setting	5	Describe the setting, locations, and relevant dates, including periods of recruitment, exposure, follow-up, and data collection (Pg6)
Participants	6	(*a*) *Cohort study*—Give the eligibility criteria, and the sources and methods of selection of participants. Describe methods of follow-up<br>*Case-control study*—Give the eligibility criteria, and the sources and methods of case ascertainment and control selection. Give the rationale for the choice of cases and controls<br>***Cross-sectional study***—Give the eligibility criteria, and the sources and methods of selection of participants (Pg6 & Figure 1)
		(*b*) *Cohort study*—For matched studies, give matching criteria and number of exposed and unexposed<br>*Case-control study*—For matched studies, give matching criteria and the number of controls per case
Variables	7	Clearly define all outcomes, exposures, predictors, potential confounders, and effect modifiers. Give diagnostic criteria, if applicable (Pg5-6)
Data sources/ measurement	8*	For each variable of interest, give sources of data and details of methods of assessment (measurement). Describe comparability of assessment methods if there is more than one group (Pg5-6)
Bias	9	Describe any efforts to address potential sources of bias (Pg11)
Study size	10	Explain how the study size was arrived at (Pg6)
Quantitative variables	11	Explain how quantitative variables were handled in the analyses. If applicable, describe which groupings were chosen and why (Pg 6)
Statistical methods	12	(*a*) Describe all statistical methods, including those used to control for confounding (Pg6)
		(*b*) Describe any methods used to examine subgroups and interactions (Pg6)
		(*c*) Explain how missing data were addressed (Pg6 – responders with missing data excluded from study)
		(*d*) *Cohort study*—If applicable, explain how loss to follow-up was addressed<br>*Case-control study*—If applicable, explain how matching of cases and controls was addressed<br>*Cross-sectional study*—If applicable, describe analytical methods taking account of sampling strategy (n/a)
		(*e*) Describe any sensitivity analyses (n/a)

For peer review only

For peer review only - http://bmjopen.bmj.com/site/about/guidelines.xhtml

Continued on next page

For peer review only

For peer review only - http://bmjopen.bmj.com/site/about/guidelines.xhtml

**Results**		
Participants	13*	(a) Report numbers of individuals at each stage of study—eg numbers potentially eligible, examined for eligibility, confirmed eligible, included in the study, completing follow-up, and analysed (Pg 7 & Figure 1)
		(b) Give reasons for non-participation at each stage (see above)
		(c) Consider use of a flow diagram (Figure 1)
Descriptive data	14*	(a) Give characteristics of study participants (eg demographic, clinical, social) and information on exposures and potential confounders (Pg 7 & Table 1)
		(b) Indicate number of participants with missing data for each variable of interest (Pg 7)
		(c) *Cohort study*—Summarise follow-up time (eg, average and total amount)
Outcome data	15*	*Cohort study*—Report numbers of outcome events or summary measures over time
		Case-control study—Report numbers in each exposure category, or summary measures of exposure
		Cross-sectional study—Report numbers of outcome events or summary measures (Pg7-8)
Main results	16	(*a*) Give unadjusted estimates and, if applicable, confounder-adjusted estimates and their precision (eg, 95% confidence interval). Make clear which confounders were adjusted for and why they were included (Pg7-8)
		(*b*) Report category boundaries when continuous variables were categorized (Pg7-8)
		(*c*) If relevant, consider translating estimates of relative risk into absolute risk for a meaningful time period (n/a)
Other analyses	17	Report other analyses done—eg analyses of subgroups and interactions, and sensitivity analyses (n/a)
**Discussion**		
Key results	18	Summarise key results with reference to study objectives (Pg 8-10)
Limitations	19	Discuss limitations of the study, taking into account sources of potential bias or imprecision. Discuss both direction and magnitude of any potential bias (Pg11)
Interpretation	20	Give a cautious overall interpretation of results considering objectives, limitations, multiplicity of analyses, results from similar studies, and other relevant evidence (Pg11)
Generalisability	21	Discuss the generalisability (external validity) of the study results (Pg9)
**Other information**		
Funding	22	Give the source of funding and the role of the funders for the present study and, if applicable, for the original study on which the present article is based (Pg2)

*Give information separately for cases and controls in case-control studies and, if applicable, for exposed and unexposed groups in cohort and cross-sectional studies.

Note: An Explanation and Elaboration article discusses each checklist item and gives methodological background and published examples of transparent reporting. The STROBE checklist is best used in conjunction with this article (freely available on the Web sites of PLoS Medicine at http://www.plosmedicine.org/, Annals of Internal Medicine at http://www.annals.org/, and Epidemiology at http://www.epidem.com/). Information on the STROBE Initiative is available at www.strobe-statement.org.

For peer review only

BMJ Open

Characterising the patient experience of diagnostic lumbar puncture in Idiopathic Intracranial Hypertension: a cross-sectional online survey.

Field	Details
Journal:	*BMJ Open*
Manuscript ID	bmjopen-2017-020445.R2
Article Type:	Research
Date Submitted by the Author:	11-Apr-2018

Field	Details
Complete List of Authors:	Scotton, William; University of Birmingham, Metabolic Neurology, Institute of Metabolism and Systems Research; University Hospitals Birmingham NHS Foundation Trust, Department of Neurology<br>Mollan, Susan; University of Birmingham, Metabolic Neurology, Institute of Metabolism and Systems Research; Birmingham Neuro-Ophthalmology Unit, Ophthalmology Department, University Hospitals Birmingham NHS Trust, Queen Elizabeth Hospital Birmingham<br>Walters, Thomas; University of Birmingham, Metabolic Neurology, Institute of Metabolism and Systems Research<br>Doughty, Sandra; Idiopathic Intracranial Hypertension UK<br>Botfield, Hannah; University of Birmingham, Metabolic Neurology, Institute of Metabolism and Systems Research; Centre for Endocrinology, Diabetes and Metabolism, Birmingham Health Partners<br>Markey, Keira; University of Birmingham, Metabolic Neurology, Institute of Metabolism and Systems Research; University Hospitals Birmingham NHS Foundation Trust, Department of Neurology<br>Yiangou, Andreas; University of Birmingham, Metabolic Neurology, Institute of Metabolism and Systems Research; University Hospitals Birmingham NHS Foundation Trust, Department of Neurology<br>Williamson, Shelley; Idiopathic Intracranial Hypertension UK<br>Sinclair, AJ; University of Birmingham, Metabolic Neurology, Institute of Metabolism and Systems Research; Centre for Endocrinology, Diabetes and Metabolism, Birmingham Health Partners

Field	Details
**Primary Subject Heading**:	Neurology
Secondary Subject Heading:	Patient-centred medicine, Medical education and training, Ophthalmology, Evidence based practice
Keywords:	Adult neurology < NEUROLOGY, Neuro-ophthalmology < NEUROLOGY, NEUROPATHOLOGY, Neuro-ophthalmology < OPHTHALMOLOGY

SCHOLARONE™
Manuscripts

Title: Characterising the patient experience of diagnostic lumbar puncture in Idiopathic Intracranial Hypertension: a cross-sectional online survey.

Authors: William J. Scotton[1,2], Susan P. Mollan[1,3], Thomas Walters[1], Sandra Doughty[4], Hannah Botfield[1,5], Keira Markey[1,2], Andreas Yiangou[1,2], *Shelly Williamson[4], *Alexandra J. Sinclair[1,5].

Institutions

1 Metabolic Neurology, Institute of Metabolism and Systems Research, College of Medical and Dental Sciences, University of Birmingham, Birmingham, B15 2TT, United Kingdom

2 Department of Neurology, University Hospitals Birmingham NHS Foundation Trust, B15 2TH, United Kingdom.

3 Birmingham Neuro-Ophthalmology Unit, Ophthalmology Department, University Hospitals Birmingham NHS Trust, Queen Elizabeth Hospital Birmingham, Birmingham, UK

4 Idiopathic Intracranial Hypertension UK, Registered Charity in England & Wales No. 1143522 & Scotland SC043294

5 Centre for Endocrinology, Diabetes and Metabolism, Birmingham Health Partners, B15 2TH, United Kingdom

*share joint senior author

Word Count Abstract: 355

Word Count Paper: 2567 (not including 67 recommendations, 111 strengths/weaknesses)

Table(s): 2

Figure(s): 3

Corresponding author:

Alexandra Sinclair, Metabolic Neurology, Institute of Metabolism and Systems Research, University of Birmingham, Edgbaston, B15 2TT, UK; a.b.sinclair@bham.ac.uk

For peer review only

Study funded by:

A.S. is funded by an NIHR Clinician Scientist Fellowship (NIHR-CS-011-028) and by the Medical Research Council, UK (MR/K015184/1).

Acknowledgments:

With thanks to Peter Nightingale (PN) (Senior Statistician, University of Birmingham), for his advice on question framing for quantitative analysis of responses, as well as in planning and interpretation of statistical analysis, and to the IIH UK charity, as well as the IIH patient group without which this study would not have been possible

Competing interests statement

None Declared.

Author's contribution to the research

William J. Scotton: Compilation of the survey results; statistical analysis; interpretation of the results and drafting the manuscript

Susan P. Mollan: Conception and design of the survey; critical review of the manuscript

Thomas Walters: Compilation of the survey results and statistical analysis

Sandra Doughty: Conception and design of the survey

Hannah Botfield Statistical analysis and interpretation of the results

Keira Markey: Interpretation off the results and drafting of the manuscript

Andreas Yiangou: Interpretation off the results and drafting of the manuscript

Shelly Williamson: Conception and design of the survey

Alexandra J. Sinclair: Conception and design of the survey; interpretation of the results and critical review of the manuscript

All authors have read and approved the final manuscript.

Data sharing statement: We are happy to share all data, including raw data if requested.

For peer review only

Abstract

Objectives

Patients with idiopathic intracranial hypertension (IIH) usually require multiple lumbar punctures during the course of their disease, and often report significant morbidity associated with the procedure. The aim of this study was to assess the patient's experience of diagnostic lumbar puncture (LP) in IIH.

Design, methods and participants

A cross-sectional study of IIH patients was conducted using an anonymous online survey, with the questions designed in collaboration with IIH UK (the UK IIH charity). Responses were collated over a two-month period from April to May 2015. Patients were asked to quantify responses using a verbal rating score (VRS) 0-10 with 0 being the minimum and 10 the maximum score.

Results

502 patients responded to the survey, of which 463 were analysed for this study. 40% of patients described severe pain during the LP (VRS ≥8), and the median pain score during the LP was 7 (VRS, IQR 5-7). The majority of patients felt they received insufficient pain relief (85%). Levels of anxiety about future LPs were high (median VRS 7, IQR 4-10), with 47% being extremely anxious (VRS ≥8). LPs performed as an emergency were associated with significantly greater pain scores compared to elective procedures (median 7, IQR 5-7 vs. 6, IQR 4-8, p=0.012). 10.7% went on to have an X-Ray guided procedure due to failure of the initial LP, and the BMI was significantly higher in this group (mean kg/m^2 40.3 vs. 35.5, p=0.001). Higher LP pain scores (VRS) were significantly associated with poorly informed patients (Spearman correlation, r=-0.32, p<0.001). Patients felt more informed when the LP was performed by a Specialist Registrar compared to a Junior Doctor (median 7 vs. 5, p=0.001) or a Consultant compared to a Junior Doctor (median 8 vs. 5, p<0.001).

For peer review only

Conclusions

This study was commissioned by the IIH patient group and is the first to document the patient experience of diagnostic lumbar punctures in IIH. It shows that the majority of these patients are experiencing significant morbidity from pain and anxiety. Patient experience of LP may be improved through changing clinical practice to include universal detailed pre-procedural information, and where possible, avoiding emergency LPs in favour of LPs booked on an elective day case unit.

Strengths and limitations of this study

- This large sample size UK survey is the first known to directly and specifically document the patient experience of diagnostic lumbar punctures in IIH, and it confirms that a significant number of these patients are experiencing morbidity from pain and anxiety related to the procedure.
- The use of an online questionnaire ensured anonymity, thus increasing the likelihood of honest reporting by patients of their subjective experiences of the procedure.
- Given the self-report nature of this study, the results may be susceptible to recall bias, thus limiting the generalisability of our findings.

For peer review only

Introduction

Idiopathic intracranial hypertension (IIH) is characterised by raised intracranial pressure (ICP) which can cause papilloedema with significant visual loss in some, as well as severe disabling headaches which significantly impact on quality of life in the majority (1)(2). The diagnostic criteria for IIH is based on an elevated lumbar puncture (LP) opening pressure (≥250 mm CSF in adults) in a properly performed lumbar puncture (3).

Many patients have multiple LPs during the disease course typically to assess disease severity, and in some cases as a therapeutic strategy. Established complications of LPs include local discomfort, low pressure headaches and more rarely infection or local haemorrhage (4). We have been made aware of an additional significant complication of LPs voiced by the patients themselves. The patients describe a very negative experience of LPs associated with anxiety, fear and pain during and after the procedure. The National charity IIH UK (Registered Charity in England and Wales no 1143522 & Scotland SCO43294) approached us with concerns about the IIH patient experience of LPs. Patient experience of spinal anaesthesia and LP has previously been studied (5)(6). However, the experience of IIH patients undergoing LP has not been evaluated. LPs are typically more technically challenging in the IIH population as over 90% of these patients are obese (3).

The aim of this study was to assess the patient experience of diagnostic LPs in IIH. We aimed to disseminate this evidence to medical professionals to increase awareness of this potential morbidity of LP in IIH patients. Furthermore, we aimed to use evidence from this study as a catalyst to drive improvements in patient care.

Material and Methods

Public and Patient Involvement

This research was initiated, designed and conducted by IIH UK, a charity that supports IIH patients and carers. The charity agreed at a Trustee meeting to design a survey to investigate the magnitude of lumbar puncture related anxiety in response to their overwhelming messages from patients. When the first survey was performed the Trustees

For peer review only

recognised that they would need help both in analysis of the data as well as asking additional questions. A further survey was therefore conducted. The clinical researchers at the University Hospitals Birmingham provided support with statistical analysis and critical review of the data.

Dissemination of the results was planned via physician and patient meetings, through medical and patient lead social media, and on the IIH UK patients' charity website.

Study Design

The cross-sectional study was conducted using an online survey. IIH UK sent a survey monkey questionnaire through social media outlets (Facebook, Twitter (@IIHUK) and IIH UK charity website (www.iih.org.uk) and allowed a two-month period from 1st April to 31st May 2015 for responses. Questionnaires were excluded if the respondents were under the age of 16 years or the survey was incomplete (missing key data fields) or uninterpretable. Anonymised data was analysed by the clinical team with input from the clinical research facility statistician (PN). As the charity board had already agreed with their members beforehand, and both surveys instructed the respondents that the information would be used to be published within the medical literature, no further ethical approval was required.

The questionnaire (see supplementary document) detailed baseline demographic details (age, weight and height), and details of the LP (emergency versus planned procedure, hospital setting, number of attempts, whether went on to have an X-Ray guided procedure and seniority of doctor performing). Data on anxiety (for the LP and future LPs), pain experienced and extent of understanding of the procedure was also collected. Patients were asked to quantify responses using a verbal rating score (VRS) 0-10 with 0 being the minimum and 10 the maximum score.

Statistical analysis

Statistical analysis was performed using SPSS versions 24 (SPSS Inc., Chicago, IL, USA) and GraphPad Prism 7 for Windows (GraphPad Software Inc., La Jolla, CA, USA). Assessment of data for normality was performed for each analysis. Normally distributed data

For peer review only - http://bmjopen.bmj.com/site/about/guidelines.xhtml

was reported using mean and standard deviation (SD), and non-normally distributed data was reported using median and interquartile range (IQR). For all comparisons of continuous variables, a non-parametric test was used due to non-normality of data distribution. For comparison of two medians Mann-Whitney U tests were used, whilst for comparison of multiple medians Kruskall-Wallis H tests were used. Spearman's rank-order correlation was used to analyse the correlation between how informed the patients were and how much pain they felt, as well as between BMI and how scared a patient was beforehand, how much pain they felt, and how anxious they felt about future LPs. For comparison of categorical variables Chi square tests were used. Values were considered statistically significant when P values were less than 0.05.

Results

Demographics

There were 502 responders to the study, of which 463 were eligible for analysis. 18 responders did not complete the survey, 11 were under the age of 16 years, and 10 gave incomplete answers or ambiguous information that could not be objectively interpreted (Figure 1). The mean age was 33 years (SD 8.9), 98.5% were female (n=456), with a mean weight of 97.4kg (SD 22.3), and a mean Body Mass Index (BMI) of 36 kg/m^2 (SD 8.3). The median number of LPs undergone since diagnosis was 4 (inter-quartile range [IQR] 1-11), though 3.1% of patients (n=15) reported more than 50 LPs. When number of LPs was adjusted to reflect length of disease, the median number of LPs per year since diagnosis was 1.3 (IQR 0.3-3.6) (Table 1).

Pain, Anxiety & Analgesia

The majority of patients indicated they were extremely scared about the imminent LP (median VRS 8, IQR 6-10), with 60% indicating a VRS ≥8 in relation to how scared they felt (Figure 2A & 2B). 40% of patients described severe pain during the LP (VRS ≥8) with a median pain score of 7 (VRS, IQR 5-8) (Figure 2A & 2C). Additionally, the majority of

For peer review only

patients felt they received insufficient pain relief (85%). Levels of anxiety about future LPs were high (median VRS 7, IQR 4-10), with 47% being extremely anxious (VRS ≥8) (Figure 2A & 2D). There was no relationship found between the pre-procedure anxiety levels and the subsequent recalled pain score of the LP.

Setting of LP and pre-procedural information

LPs were predominantly performed in the emergency setting (72%), as opposed to as an elective planned procedure on day-case unit. Importantly the LPs performed in the emergency setting were associated with significantly greater pain scores compared to elective procedures (VRS median 7, IQR 5-7 vs 6, IQR 4-8 respectively, p=0.012).

Only 37% of patients felt well informed about LP pre-procedure (VRS ≥8); 27% felt poorly informed (VRS 0-3), and 7% did not feel they were informed at all (VRS 0). Higher LP pain scores (VRS) were significantly associated with patients being poorly informed (Spearman correlation, r=-0.32, p<0.001) (Figure 3A). Patients felt better informed if they had an elective planned LP compared to a procedure in the emergency setting (median 7, IQR 5-10 versus median 6, IQR 5-10, p=0.011).

Difficulty of LP and need for X-ray guided procedure

47% of patients had 2 or more doctors attempt their LP (median 1, IQR 1-2) while 45% had greater than 3 attempts (number of times needle inserted) before success. 10.7% went on to have an X-Ray guided procedure due to failure of the initial LP, and the BMI was significantly higher in this group (mean kg/m^2 40.3 vs. 35.5, p=0.001) (Figure 3B). There was only a weak correlation between BMI and how scared a patient was beforehand, how much pain they felt, and how anxious they felt about future LPs (Spearman r = 0.17, 0.17, 0.17 respectively, p<0.001 for all). Compared to those that had normal LPs, the patients having X-Ray guided procedures felt less informed (VRS median 3 vs 6, p=0.002), suffered more pain (VRS median 8 vs 7, p=0.004), and were more anxious about future LPs (VRS median 9 vs 7, p=0.003).

For peer review only - http://bmjopen.bmj.com/site/about/guidelines.xhtml

Grade of Doctor performing LP

Table 2 shows the number of LP attempts by grade of doctor performing the LP. Patients felt more informed when the LP was performed by a Specialist Registrar (SpR) compared to a Junior doctor (median VRS 7 vs 5, p=0.001) or a Consultant compared to a Junior doctor (median VRS 8 vs 5, p< 0.001), though there was no significant difference in the pain scores reported. They also suffered from less severe post-LP headaches (SpR vs Junior median VRS 7 vs 8, p<0.001, Consultant vs Junior median VRS 6.5 vs 8, p=0.003) (Figure 3C), and length of post-LP headache (SpR vs Junior median days 3 vs 6, p=0.02, Consultant vs Junior median days 4 vs 6, p=0.9) (Figure 3D).

Discussion

This is the first study, to the best of our knowledge, to document the patient experience of diagnostic lumbar punctures in IIH. It has shown that a number of patients are recalling significant pain and anxiety. This morbidity is associated with inadequate pre-procedural information, the environment the LP is performed in (emergency setting being associated with increased pain), and the seniority of the doctor performing the LP.

Anaesthetists have long recognised the importance of the patient experience of spinal anaesthesia as an outcome measure and an indicator of quality of care (5)(6)(7). This is reflected in the high satisfaction levels patients report with the procedure (96-97%), which is in stark contrast to the feedback here. The differences between the anaesthetic population and the IIH patient group may be related to the procedure being technically more challenging due to the patient's high BMI, the procedure happening as an emergency and some having multiple LPs during the course of their IIH. It may also be due to anaesthetists having better technical skills due to performing the procedure more often than the doctors (often non-neurologists) performing the LPs in the emergency setting, in addition to more closely supervised and rigorous training.

This was a large sample size study (463 responders) where patients could respond anonymously, thus increasing the likelihood of honest reporting of their subjective

For peer review only

experiences of the procedure. This cohort reports the LP experience as negative with 40% of patients experiencing severe pain (VRS ≥8) during the procedure, 85% saying they did not receive adequate analgesia and 47% stating they were extremely anxious (VRS ≥8) about future LPs.

The majority of the group did not feel they received adequate pre-procedural information, with 63% not feeling well informed (VRS <8), and 7% saying that they were not informed at all (VRS =0). Patients who were less informed experienced more pain during the procedure. Although all patients will have undergone a consent process in the UK, this data highlights the variable quality of the information disseminated by the physician to the patient. Current practices for informing patients about LP are likely to be highly variable across the UK. This study highlights a key area where simple changes in clinical practice to ensure all patients are provided with detailed pre-procedure information could facilitate improved patient care.

Environment also had a bearing on the patient experience with 72% of LPs being performed in the emergency setting; this was associated with the patient feeling less informed, and reporting significantly higher pain scores, compared to an elective procedure on a day-case unit. The high portion of the respondents who had an LP performed as part of an emergency admission in this study likely reflects the UK health care services and clinical practice where patients with a flare up in IIH symptoms are typically initially seen in the accident and emergency department for initial evaluation and often have a LP as part of their evaluation here, or on the acute medical unit. As the study was not designed to determine the clinical indications for the LP in each case no further inference can be made here. Typically LPs performed in the emergency setting may be conducted by junior physicians, with less experience in conducting LPs than a speciality trained neurologist or anaesthetist. This may be a factor contributing to the poorer outcomes from LPs performed in the emergency setting.

Optimisation of the environment for the patient undergoing LP could therefore positively affect their outcome. The day case environment may provide access to doctors adequately trained in performing LPs as well as a less time-pressurised environment. Time to reflect on

For peer review only

the procedure and read pre-procedural information, may help improve the overall experience. Although diverting IIH patients away from the emergency setting would likely benefit the majority, it may not be practical for a minority of papilloedema cases where there is progressive or rapid loss of visual function, and the need for acute diagnosis.

The study also suggests that there is also scope for improving our technical skills in LP, as 85% of our cohort stated that they did not receive adequate analgesia, with 45% undergoing greater than 3 attempts (defined as the needle being fully withdrawn between attempts), and 47% having 2 or more doctors attempt the LP. When the LP was performed by a doctor more junior than registrar (30% of the time), the patients felt less informed, and reported more serve and longer lasting post-LP headaches. We acknowledge that the grade of doctor performing the LP may not be accurately recalled by the patient and maybe more of a reflection of the patient's confidence in the doctor. However diverting LPs into the day-case setting would provide an opportunity where the junior doctors could be appropriately supervised and trained, which has been shown to increase their ability to perform the procedure (8).

In this cohort 10.7% of patients reported having an x-ray guided procedure due to failure of the initial LP, with the BMI being significantly higher in this group (mean 40.3kg/m^2 vs. 35.5kg/m^2, p=0.001). This finding is in keeping with a recent study which showed a strong correlation between BMI and procedure failure, with half of the failed LPs occurring in patients with a BMI greater than 35kg/m^2 (9). The inability to palpate landmarks in obese patients is likely to be a significant driver of this correlation (10). The x-ray guided LP group felt less informed, reported more pain, and were more likely to feel anxious about future LPs; findings most likely due to the number of failed attempts before the x-ray guided procedure. The growing evidence base for use of ultrasound guidance, particularly in patients with a higher BMI and absence of landmarks (11), suggests that its use in the IIH patient cohort may increase the success rate of the initial LP and decrease the number that require x-ray guided procedures.

For peer review only

For clinical care a positive experience of a diagnostic LP will positively impact on the patient's future engagement with healthcare services, whilst in IIH research LP experience affects recruitment to clinical trials (12); it is therefore critical that clinicians optimise patient care.

Limitations

Given the self-report nature of this study the results are likely susceptible to recall bias. Interpretation of some of the study questions is problematic: for example, for questions such as the number of attempts for a lumbar puncture and the seniority of the doctor performing the procedure, the respondents may not accurately know the answer.

Conclusion

There has been a growing consensus in recent years that if healthcare services are to better deliver patient-centred care then research needs to be more reflective of patients' needs and concerns (13)(14). This study was commissioned by the IIH patient group and is the first to document the patient experience of diagnostic lumbar punctures in IIH. It documents experiences of significant pain and anxiety associated with both inadequate pre-procedural information and the setting the LP is performed in. The study suggests a number of practical steps that may improve the patient experience of LPs.

Recommendations for improving patient experience of diagnostic LP in IIH
• Providing enhanced pre-procedural information.<br>• Where possible, diverting emergency department LPs to elective procedures on dedicated day case units.<br>• Simulation training for doctors and specialist nurses to develop appropriate technical (including ultrasound guidance) and human factor skills (such as communication, empathy and leadership) for performing LPs in a technically difficult patient cohort.

For peer review only

- Implementing widespread patient reported outcome measures for LPs to guide the need for service improvements and training needs.

Bibliography

1. Mollan SP, Ali F, Hassan-Smith G, et al. Evolving evidence in adult idiopathic intracranial hypertension: pathophysiology and management. J Neurol Neurosurg Psychiatry. 2016;87(9):982-92
2. Mulla Y, Markey KA, Woolley RL, et al. Headache determines quality of life in idiopathic intracranial hypertension. J Headache Pain. 2015;16:521
3. Markey KA, Mollan SP, Jensen RH, et al. Understanding idiopathic intracranial hypertension: mechanisms, management, and future directions. Lancet Neurol. 2016;15(1):78-91.
4. Wright BLC, Lai JTF, Sinclair AJ. Cerebrospinal fluid and lumbar puncture: A practical review. J Neurol. 2012;259(8):1530–45.
5. Rhee WJ, Chung CJ, Lim YH, et al. Factors in patient dissatisfaction and refusal regarding spinal anesthesia. Korean J Anesthesiol. 2010;59(4):260-4
6. Dharmalingam TK, Ahmad Zainuddin NA. Survey on maternal satisfaction in receiving spinal anaesthesia for caesarean section. Malaysian J Med Sci. 2013;20(3):51–4.
7. Wu CL, Naqibuddin M;, Fleisher LA. Measurement of patient satisfaction as an outcome of regional anesthesia and analgesia: a systematic review. Reg Anesth Pain Med. 2001;26(3):196-208.
8. Sweeney M, Al-Diwani A, Hadden R. Improving the safety and efficiency of outpatient lumbar puncture service. BMJ Qual Improv Rep. 2016;25:5(1).
9. Edwards C, Leira EC, Gonzalez-Alegre P. Residency training: a failed lumbar puncture is more about obesity than lack of ability. Neurology. 2015 Mar 10;84(10):e69-72.
10. Stiffler KA, Jwayyed S, Wilber ST, et al. The use of ultrasound to identify pertinent

For peer review only

landmarks for lumbar puncture. Am J Emerg Med. 2007;25(3):331-4.

11. Soni NJ, Franco-Sadud R, Schnobrich D, et al. Ultrasound guidance for lumbar puncture. Neurol Clin Pract. 2016;6(4):358-368.

12. Ball AK, Howman A, Wheatley K, et al. A randomised controlled trial of treatment for idiopathic intracranial hypertension. J Neurol. 2011;258(5):874-81.

13. Chalmers I, Bracken MB, Djulbegovic B, et al. How to increase value and reduce waste when research priorities are set. Lancet. 2014;383(9912):156-65.

14. Entwistle V, Calnan M, Dieppe P. Consumer involvement in setting the health services research agenda: persistent questions of value. J Health Serv Res Policy. 2008;13(3):76–81

Competing interests statement

None Declared.

Author's contribution to the research

William J. Scotton Compilation of the survey results; statistical analysis; interpretation of the results and drafting the manuscript

Susan P. Mollan Conception and design of the survey; critical review of the manuscript

Thomas Walters Compilation of the survey results and statistical analysis

Sandra Doughty Conception and design of the survey

Hannah Botfield Statistical analysis and interpretation of the results

Keira Markey: Interpretation off the results and drafting of the manuscript

Andreas Yiangou: Interpretation off the results and drafting of the manuscript

Shelly Williamson Conception and design of the survey

Alexandra J. Sinclair Conception and design of the survey; interpretation of the results and critical review of the manuscript

All authors have read and approved the final manuscript.

For peer review only

Figure 1: Baseline characteristics of eligible responders

Table 1: Baseline characteristics of eligible responders

Variable	No. (%) n=463
Age, mean, years (SD)	32.9 (8.9)
Female sex	456 (98.5%)
Weight, mean, kg (SD)	97.2 (22.5)
BMI, mean (SD)	36.0 (8.3)
LPs since diagnosis, median (IQR)	4 (1-11)
LPs per year since diagnosis, median (IQR)	1 (1-4)

Figure 2: Patients' expectations and experience of LP. (A) Median VRS (0-10, IQR) for how scared patient was before LP, how painful the LP was and how anxious they were about future LPs. (B) Number of patients that were mildly (0-3), moderately (4-7), or very scared (8-10) before having an LP. (C) Number of patients that experienced mild (VRS 0-3), moderate (VRS 4-7) or severe (VRS 8-10) pain during the LP. (D) Number of patients that were mildly (VRS 0-3), moderately (VRS 4-7) or very anxious (VRS 8-10) about future LPs. [VRS 0=minimum and 10 maximal score]

Figure 3: X-Ray guided LPs, and relationship of pre-procedural information and grade of doctor performing LP to patient experience. (A) For all patients surveyed, association between how well-informed patient was before LP, and how painful LP was (median VRS, IQR, min-max). (B) BMI (median, IQR) and association with whether patient had X-Ray

guided LP. (C) Grade of doctor performing LP and duration of post-LP headache (days, median, IQR). (D) Grade of doctor performing LP and severity of post-LP headache. ns p>0.05, * p ≤0.05, ** p≤0.01, *** p≤0.001, [VRS 0=minimum and 10=maximal score].

Table 2: Number of LP attempts by grade of doctor

**Number of LP attempts**	**Grade of Doctor (% of total patients [n=463])**				
	Unknown	Junior	Registrar	Consultant	**Total**
1-3	13.0%	9.1%	15.1%	11.0%	**48.2%**
4-6	4.8%	5.2%	6.3%	3.9%	**20.1%**
7-9	2.6%	1.9%	3.0%	1.7%	**9.3%**
10-14	0.6%	1.7%	1.3%	0.4%	**4.1%**
15-19	0.4%	1.1%	0.4%	0.0%	**1.9%**
20+	0.6%	1.1%	0.4%	0.2%	**2.4%**
Unknown	4.5%	1.1%	3.2%	5.2%	**14.0%**
**Total**	**26.6%**	**21.2%**	**29.8%**	**22.5%**	**100%**

iew only

For peer review only - http://bmjopen.bmj.com/site/about/guidelines.xhtml

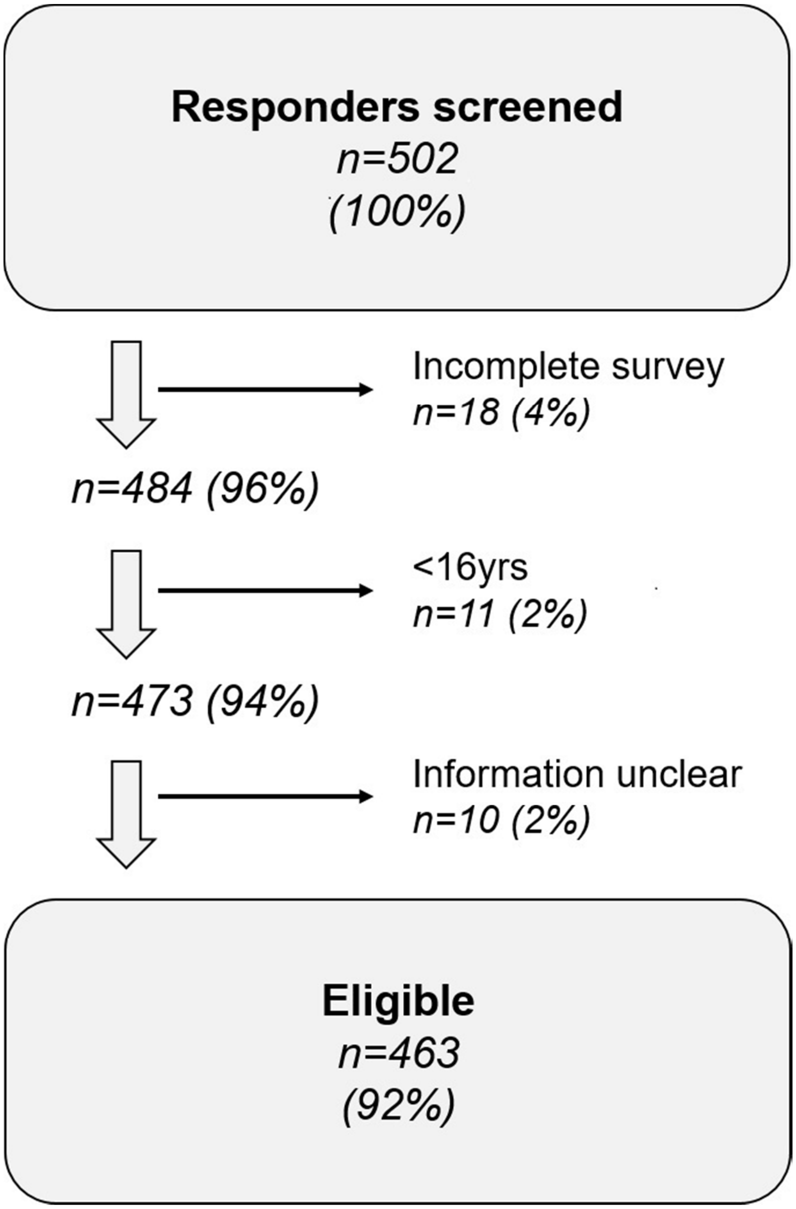

Figure 1: Baseline characteristics of eligible responders.

57x87mm (300 x 300 DPI)

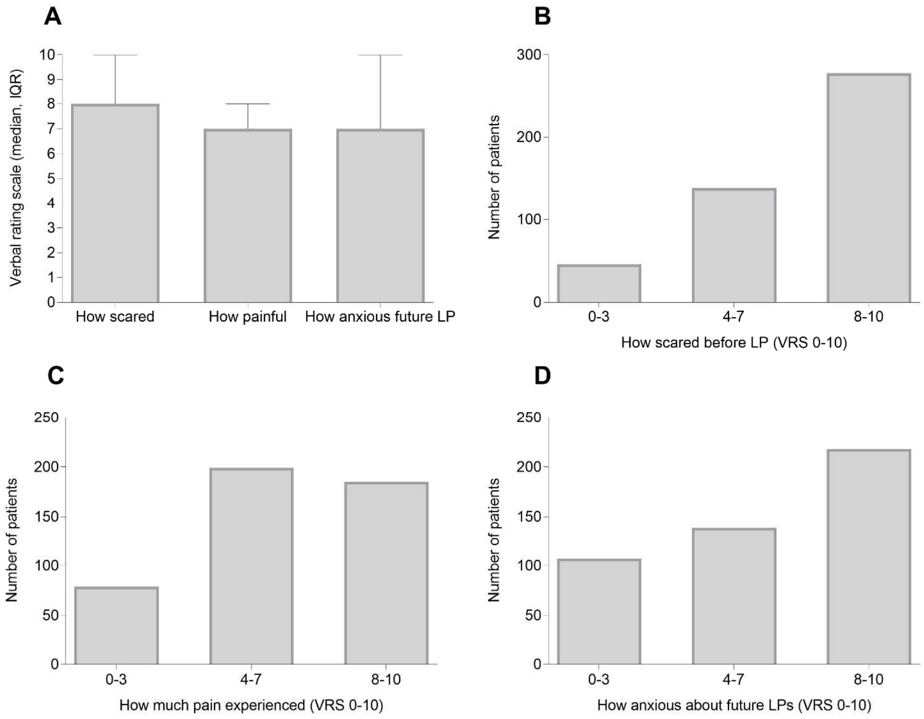

Figure 2: Patients' expectations and experience of LP. (A) Median VRS (0-10, IQR) for how scared patient was before LP, how painful the LP was and how anxious they were about future LPs. (B) Number of patients that were mildly (0-3), moderately (4-7), or very scared (8-10) before having an LP. (C) Number of patients that experienced mild (VRS 0-3), moderate (VRS 4-7) or severe (VRS 8-10) pain during the LP. (D) Number of patients that were mildly (VRS 0-3), moderately (VRS 4-7) or very anxious (VRS 8-10) about future LPs. [VRS 0=minimum and 10 maximal score].

282x212mm (300 x 300 DPI)

For peer review only - http://bmjopen.bmj.com/site/about/guidelines.xhtml

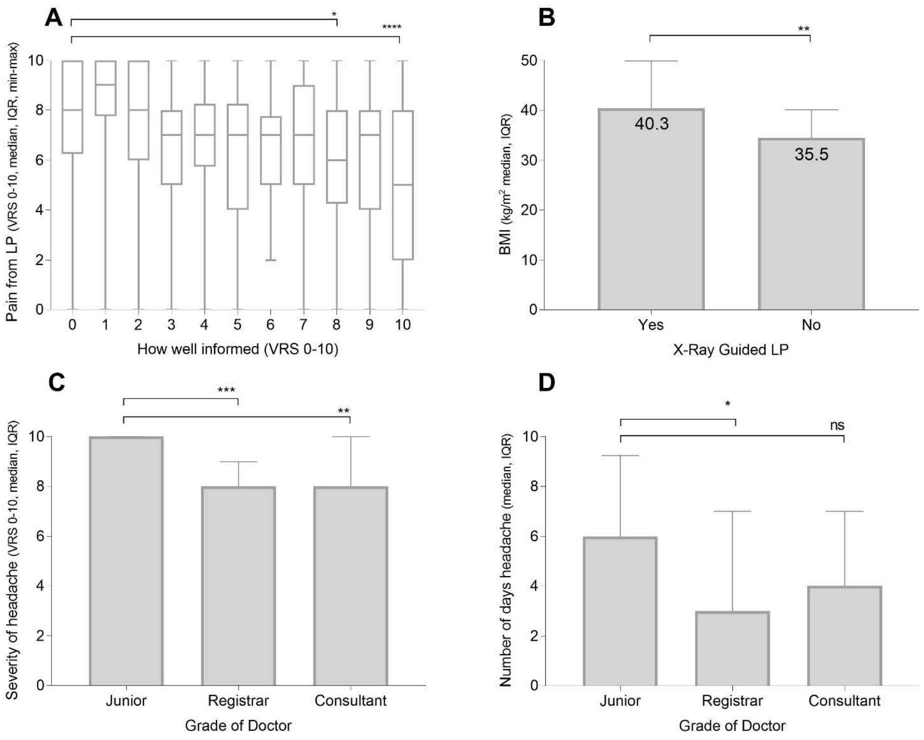

Figure 3: X-Ray guided LPs, and relationship of pre-procedural information and grade of doctor performing LP to patient experience. (A) For all patients surveyed, association between how well-informed patient was before LP, and how painful LP was (median VRS, IQR, min-max). (B) BMI (median, IQR) and association with whether patient had X-Ray guided LP. (C) Grade of doctor performing LP and duration of post-LP headache (days, median, IQR). (D) Grade of doctor performing LP and severity of post-LP headache. ns p>0.05, * p ≤0.05, ** p≤0.01, *** p≤0.001, [VRS 0=minimum and 10=maximal score].

280x217mm (300 x 300 DPI)

IIHUK Online Questionnaire

Demographics

1. How old were you at time of diagnostic LP?
2. What was date of your first lumbar puncture? (Do not worry if you cannot remember the exact day, but please specify month and year)
3. Since your first diagnostic LP, how many LPs have you had? (Please approximate if not sure of the exact number)
4. What is your height (metres)?
5. What is your weight (kg)?

Details of lumbar puncture

1. Was your first admission regarding your LP planned (you were sent an appointment by your doctor) or as an emergency?
2. Where in the hospital did the first LP take place? (Emergency department, Ward or In Theatre)
3. How many different doctors attempted your diagnosing LP? (i.e how many different doctors tried inserting a needle)
4. How many attempts were made to get a diagnostic LP? (An attempt being defined as a needle being inserted)
5. If the initial LP failed to get a reading did you go on to have it done in theatre under X-ray guidance?

Patient experience of lumbar puncture

1. How frightened were you about the thought of the LP before the procedure on a scale 0-10? (0 being not frightened at all, 10 being the most scared you’ve ever felt)
2. How well do you feel you were informed about the procedure prior to the LP on a scale 0-10? (0 being not informed, 10 being fully informed)
3. Was adequate analgesia (i.e. local anaesthetic) used in your diagnosing LP?

For peer review only

4. How much pain did you experience during the LP on a scale of 0-10? (0 being pain free, 10 being worst pain ever experienced)
5. On a scale of 0-10, how anxious do you feel about having LPs in the future? (0 being not anxious, 10 being the most anxious you've ever felt)

For peer review only

STROBE Statement—checklist of items that should be included in reports of observational studies

	Item No	Recommendation
**Title and abstract**	1	(*a*) Indicate the study's design with a commonly used term in the title or the abstract (Pg1)
		(*b*) Provide in the abstract an informative and balanced summary of what was done and what was found (Pg 3-4)
**Introduction**		
Background/rationale	2	Explain the scientific background and rationale for the investigation being reported (Pg5)
Objectives	3	State specific objectives, including any prespecified hypotheses (Pg5)
**Methods**		
Study design	4	Present key elements of study design early in the paper (Pg5-6)
Setting	5	Describe the setting, locations, and relevant dates, including periods of recruitment, exposure, follow-up, and data collection (Pg6)
Participants	6	(*a*) *Cohort study*—Give the eligibility criteria, and the sources and methods of selection of participants. Describe methods of follow-up<br>*Case-control study*—Give the eligibility criteria, and the sources and methods of case ascertainment and control selection. Give the rationale for the choice of cases and controls<br>***Cross-sectional study***—Give the eligibility criteria, and the sources and methods of selection of participants (Pg6 & Figure 1)
		(*b*) *Cohort study*—For matched studies, give matching criteria and number of exposed and unexposed<br>*Case-control study*—For matched studies, give matching criteria and the number of controls per case
Variables	7	Clearly define all outcomes, exposures, predictors, potential confounders, and effect modifiers. Give diagnostic criteria, if applicable (Pg5-6)
Data sources/ measurement	8*	For each variable of interest, give sources of data and details of methods of assessment (measurement). Describe comparability of assessment methods if there is more than one group (Pg5-6)
Bias	9	Describe any efforts to address potential sources of bias (Pg11)
Study size	10	Explain how the study size was arrived at (Pg6)
Quantitative variables	11	Explain how quantitative variables were handled in the analyses. If applicable, describe which groupings were chosen and why (Pg 6)
Statistical methods	12	(*a*) Describe all statistical methods, including those used to control for confounding (Pg6)
		(*b*) Describe any methods used to examine subgroups and interactions (Pg6)
		(*c*) Explain how missing data were addressed (Pg6 – responders with missing data excluded from study)
		(*d*) *Cohort study*—If applicable, explain how loss to follow-up was addressed<br>*Case-control study*—If applicable, explain how matching of cases and controls was addressed<br>*Cross-sectional study*—If applicable, describe analytical methods taking account of sampling strategy (n/a)
		(*e*) Describe any sensitivity analyses (n/a)

For peer review only - http://bmjopen.bmj.com/site/about/guidelines.xhtml

Continued on next page

For peer review only

**Results**		
Participants	13*	(a) Report numbers of individuals at each stage of study—eg numbers potentially eligible, examined for eligibility, confirmed eligible, included in the study, completing follow-up, and analysed (Pg 7 & Figure 1)
		(b) Give reasons for non-participation at each stage (see above)
		(c) Consider use of a flow diagram (Figure 1)
Descriptive data	14*	(a) Give characteristics of study participants (eg demographic, clinical, social) and information on exposures and potential confounders (Pg 7 & Table 1)
		(b) Indicate number of participants with missing data for each variable of interest (Pg 7)
		(c) *Cohort study*—Summarise follow-up time (eg, average and total amount)
Outcome data	15*	*Cohort study*—Report numbers of outcome events or summary measures over time
		Case-control study—Report numbers in each exposure category, or summary measures of exposure
		Cross-sectional study—Report numbers of outcome events or summary measures (Pg7-8)
Main results	16	(*a*) Give unadjusted estimates and, if applicable, confounder-adjusted estimates and their precision (eg, 95% confidence interval). Make clear which confounders were adjusted for and why they were included (Pg7-8)
		(*b*) Report category boundaries when continuous variables were categorized (Pg7-8)
		(*c*) If relevant, consider translating estimates of relative risk into absolute risk for a meaningful time period (n/a)
Other analyses	17	Report other analyses done—eg analyses of subgroups and interactions, and sensitivity analyses (n/a)
**Discussion**		
Key results	18	Summarise key results with reference to study objectives (Pg 8-10)
Limitations	19	Discuss limitations of the study, taking into account sources of potential bias or imprecision. Discuss both direction and magnitude of any potential bias (Pg11)
Interpretation	20	Give a cautious overall interpretation of results considering objectives, limitations, multiplicity of analyses, results from similar studies, and other relevant evidence (Pg11)
Generalisability	21	Discuss the generalisability (external validity) of the study results (Pg9)
**Other information**		
Funding	22	Give the source of funding and the role of the funders for the present study and, if applicable, for the original study on which the present article is based (Pg2)

*Give information separately for cases and controls in case-control studies and, if applicable, for exposed and unexposed groups in cohort and cross-sectional studies.

Note: An Explanation and Elaboration article discusses each checklist item and gives methodological background and published examples of transparent reporting. The STROBE checklist is best used in conjunction with this article (freely available on the Web sites of PLoS Medicine at http://www.plosmedicine.org/, Annals of Internal Medicine at http://www.annals.org/, and Epidemiology at http://www.epidem.com/). Information on the STROBE Initiative is available at www.strobe-statement.org.

For peer review only
